# Three-month biomarker changes after spirulina with or without nursing-led dietary management in older adults with coronary artery disease: a prospective nonrandomized comparative study

**DOI:** 10.3389/fmed.2026.1910826

**Published:** 2026-08-14

**Authors:** Juan Zhang, Qingyi Xu, Yinxia Dou, Jing Chen, Qing Li

**Affiliations:** 1 Department of Nursing, Zhengzhou Shuqing Medical College, Zhengzhou, Henan, China; 2 Department of Emergency, Qingdao No. 5 People's Hospital, Qingdao, Shandong, China; 3 Department of Surgery, Henan Armed Police Corps Hospital, Zhengzhou, Henan, China; 4 Department of Outpatient, Weinan Central Hospital, Weinan, Shaanxi, China

**Keywords:** coronary artery disease, dietary control, endothelial function, homocysteine, lipid profile, nursing, observational cohort study, spirulina

## Abstract

**Background:**

Spirulina has been studied as a low-cost nutritional supplement, but evidence in older adults with coronary artery disease is limited and nonrandomized comparisons are vulnerable to healthy-adherer and treatment-selection bias.

**Methods:**

We conducted a single-center, prospective, nonrandomized interventional comparison of conventional nursing care, spirulina plus routine dietary guidance, and structured nursing-led dietary management plus spirulina (88 participants per group). Group assignment was fixed at enrolment. Ten nutritional, lipid, homocysteine and endothelial biomarkers were assessed at baseline and 3 months; first major adverse cardiovascular events (MACE) were followed for 18 months. Because no single biomarker was prospectively designated as primary, all biomarker analyses were treated as exploratory and false-discovery-rate correction was applied. Reviewer-requested reanalysis used baseline-adjusted regression with measured treatment-selection variables, generalized-overlap weighting as a sensitivity analysis, Kaplan-Meier and Aalen-Johansen estimators, and a parsimonious Cox model. Further sensitivity analyses restricted the cohort to the window during which all three groups were recruiting concurrently and to recipients of a single product batch.

**Results:**

Paired biomarker data were available for 259 participants. Compared with control, adjusted 3-month differences for spirulina and diet plus spirulina were, respectively, −0.12 mmol/L (95% CI −0.20 to −0.04) and −0.25 mmol/L (−0.33 to −0.17) for LDL cholesterol; −0.52 μmol/L (−0.74 to −0.31) and −1.05 μmol/L (−1.29 to −0.81) for homocysteine; and +0.89 points (+0.59 to +1.19) and +1.60 points (+1.26 to +1.95) for Mini Nutritional Assessment score. All control-group contrasts remained significant after false-discovery-rate correction, and estimates were essentially unchanged when analysis was restricted to the concurrent recruitment window. First MACE occurred in 24, 18 and 16 participants. Adjusted hazard ratios were 0.74 (0.40–1.37) and 0.62 (0.33–1.17); confidence intervals included no difference.

**Conclusions:**

Spirulina-containing strategies were associated with modestly more favorable 3-month biomarker profiles, with larger differences for the combined nursing-led dietary programme. The nonrandomized design, incomplete covariate balance and absence of a dietary-management-only arm preclude causal or synergistic interpretation. The 18-month MACE findings are exploratory and inconclusive.

## Introduction

Contemporary secondary prevention of coronary artery disease combines antithrombotic and lipid-lowering therapy with smoking cessation, physical activity, weight management, and a cardioprotective dietary pattern (1, 2). Older patients often face additional nutritional problems, including poor appetite, multimorbidity, polypharmacy and dependence on family members for meal preparation. The Mini Nutritional Assessment is widely used to characterize nutritional risk in this population (3).

Elevated homocysteine is associated with vascular risk, but randomized evidence has shown that lowering homocysteine with B vitamins does not necessarily reduce cardiovascular events (4, 5). Mechanistic claims therefore require particular caution. Although spirulina is sometimes marketed as a source of vitamin B12, most corrinoid in Arthrospira products is pseudovitamin B12, which is not considered bioavailable to humans (6, 7). Any observed homocysteine change should not be attributed to cobalamin unless active cobalamin exposure and participant B-vitamin status have been directly established.

Small trials and meta-analyses have reported favorable changes in lipid concentrations after spirulina supplementation, but the magnitude and certainty of benefit vary across populations and formulations (8–11). Structured nursing-led dietary management may improve implementation of sodium, fat, fiber, and protein targets; however, a combined spirulina-plus-dietary programme cannot establish an independent dietary effect or biological synergy without a dietary-management-only arm.

We therefore re-evaluated a prospective three-group clinical study in older adults with coronary artery disease. The principal objective was to estimate adjusted between-group differences in 3-month nutritional, lipid, homocysteine and endothelial biomarkers. An 18-month MACE analysis was retained as an exploratory clinical outcome. The present revision was rebuilt from the supplied participant-level hospital dataset, exact elapsed event and censoring times, treatment-selection variables, laboratory-quality-control records and adherence records.

## Materials and methods

### Study design, setting and reporting

This was a single-center, prospective, nonrandomized interventional comparative study conducted at Weinan Central Hospital between June 2020 and June 2023. Investigators supplied a standardized spirulina regimen, and a dedicated nursing team delivered the structured dietary programme; the design is therefore not described as an observational cohort. Reporting was informed by the STROBE and TREND statements (12, 13). The study was conducted in accordance with the Declaration of Helsinki and was approved by the Ethics Committee of Weinan Central Hospital having approval number SZK-20201H-098.

### Participants, time zero and group assignment

Eligible patients were aged 60 years or older and had clinically diagnosed coronary artery disease. Exclusion criteria were recent use of B vitamins, folate or other agents known to materially affect homocysteine; severe hepatic, renal or haematologic disease; active malignancy, infection or rheumatic disease; or incomplete baseline data. Written informed consent was reported as having been obtained before enrolment.

Time zero was the enrolment date for every participant. The attending clinical team and patient selected one of three strategies before outcomes were assessed: conventional nursing care with routine dietary guidance (control); routine dietary guidance plus spirulina; or structured nursing-led dietary management plus spirulina. The selection process reflected supplement willingness, family support and availability of the structured nursing programme, together with clinical and social considerations. These variables, education, socioeconomic status, health literacy, baseline diet quality, medication adherence and calendar period were included in the reanalysis because they were recorded and directly related to treatment selection.

The protocol used quota-limited enrolment of 88 participants per group. The supplied enrolment file records quota closure on 22 November 2022 for control, 31 January 2023 for spirulina and 9 June 2023 for diet plus spirulina. The screening log contains 342 patients formally screened while the relevant group quota was open; it does not enumerate otherwise eligible patients encountered after a quota closed. This limitation may have introduced selection bias and is reported explicitly. Recruitment was not synchronous across the three groups, because each quota closed as soon as it filled. All three groups were open concurrently between June 2020 and November 2022, during which 88 control, 81 spirulina and 72 combined-intervention participants were enrolled. A further 7 spirulina and 16 combined-intervention participants were enrolled after the control quota had closed and therefore had no contemporaneously recruited control participants. The complete enrolment timeline by group is reported in Supplementary Table S13 and displayed in Supplementary Figure S6, and a sensitivity analysis restricted to the concurrent recruitment window was added during revision for every biomarker and time-to-event outcome.

### Interventions and adherence

Control participants received conventional nursing and routine dietary advice. Participants assigned to spirulina received routine guidance plus 2.1 g/day oral Arthrospira biomass for 3 months. The supplied pharmacy certificate describes one 700-mg tablet after each main meal; the earlier description of two 350-mg tablets per dose was removed. Participants in the combined group received the same spirulina regimen and an individualized nursing-led programme addressing sodium, fat, cholesterol, protein, carbohydrate, fiber, vegetables and fruit. Menus were implemented at home with family involvement. Process targets and checklist completion are detailed in Supplementary Tables S1 and S7.

The product was Spirulina 700 (Nutraceutical Manufacturing Co., China). Three sequential manufacturing batches were dispensed across the enrolment window. Batch A (lot NIS21090450-SRL-09), the batch described in the previous version of this report, was manufactured in January 2020, carried an expiry date of 31 December 2022, and supplied 108 participants dispensed between June 2020 and September 2022. Batch B (lot SIM-SP-B12-20220915-02, expiry 30 June 2024) supplied 60 participants dispensed between October 2022 and February 2023. Batch C (lot SIM-SP-B12-20230220-01, expiry 31 December 2024) supplied 8 participants dispensed between March and June 2023. Each participant received a single 3-month supply at the time of dispensing, so the latest date on which any Batch A tablet could have been consumed was December 2022, which lies within the labeled shelf life; no participant received or consumed product after its expiry date, and no protocol deviation for expired product was recorded. Lot numbers, expiry dates, dispensing periods, residual product-use coverage and participants supplied are tabulated by batch in Supplementary Tables S12 and S13 and shown graphically in Supplementary Figure S6. Shelf life is used here in its conventional sense, namely the period during which the product is expected to remain within its release specification when stored under the labeled conditions (14). Product identity was confirmed by appearance, light microscopy and manufacturer identity testing, and a signed certificate of analysis was retrieved and matched to each of the three dispensed lots. Heavy metals were reported below the applicable food-supplement limits, and total aerobic count, yeasts and molds, coliforms, Salmonella species, and Staphylococcus aureus were tested. All three certificates record an unfortified product with heavy metals below the applicable food-supplement limits and satisfactory microbiological results. Quantitative corrinoid profiling by LC-MS/MS was performed for Batch A, which reported total corrinoids of 1.18 μg/g, predominantly pseudovitamin B12 (1.07 μg/g) with little authentic cobalamin (0.04 μg/g); the Batch B and Batch C certificates confirmed the same unfortified specification but did not repeat the LC-MS/MS corrinoid assay. Tablet counts and nursing records were used to estimate 3-month adherence. After month 3, spirulina continuation was optional and was recorded at months 6, 12, and 18; baseline group assignment was not changed.

### Outcomes and laboratory measurements

The 3-month outcomes were albumin, total protein, 18-item Mini Nutritional Assessment score, total cholesterol, triglycerides, HDL cholesterol, LDL cholesterol, homocysteine, nitric-oxide metabolites, and endothelin-1. Fasting samples were obtained at baseline and 3 months. Serum albumin, total protein, total cholesterol, triglycerides, HDL cholesterol, LDL cholesterol, and homocysteine were measured on a Roche cobas c 311 automated clinical-chemistry analyser (Roche Diagnostics; material no. 04826876001) using ALB2 Albumin Gen.2 (bromocresol green; material no. 03183688122), TP2 Total Protein Gen.2 (biuret; 03183734190), CHOL2 Cholesterol Gen.2 (03039773190), TRIGL Triglycerides (20767107322), HDLC4 HDL-Cholesterol Gen.4 (07528566190), LDL-Cholesterol plus 2nd generation, and HCYS Homocysteine Enzymatic Assay (enzymatic cycling; 05385415190). Nitric-oxide metabolites were measured with the Cayman Chemical Nitrate/Nitrite Colorimetric Assay Kit (item no. 780001; nitrate-reductase/Griess method, read at 540–550 nm) and endothelin-1 with the R&D Systems/Bio-Techne Endothelin-1 Quantikine ELISA Kit (catalog no. DET100), both read on a Thermo Scientific Multiskan FC microplate reader (catalog no. 51119000). Manufacturer-assigned calibrators were used, and two levels of internal quality-control material were analyzed each day, with results accepted only when they fell within the laboratory's predefined control limits. Assay ranges, imprecision and batch records are reported in Supplementary Table S10, and instrument, reagent and kit catalog identifiers in Supplementary Table S10a.

The clinical outcome was first MACE, defined as myocardial infarction, ischaemic stroke or cardiac death, from enrolment through day 540. Exact elapsed event or censoring days were used. Non-cardiac death was treated as a competing event. Serious adverse events were defined according to ICH E2A as death, a life-threatening event, initial or prolonged hospitalization, persistent or substantial disability, congenital anomaly, or another medically important event requiring intervention (15). Consistent with current guidance on the reporting of harms, serious events are counted in two distinct metrics that are reported separately throughout: the number of participants experiencing at least one qualifying event, and the number of individual event episodes (16). The two metrics differ because a participant may experience more than one qualifying episode during 18 months of follow-up and may contribute to both the MACE and the non-MACE category. Event severity was graded on a five-point scale: grade 1, mild with no intervention required; grade 2, moderate requiring minimal treatment; grade 3, severe or medically significant; grade 4, life-threatening; and grade 5, death. Non-serious treatment-emergent events were collected from enrolment through month 3, and serious adverse events through day 540. Laboratory-safety criteria were alanine or aspartate aminotransferase greater than three times the laboratory upper limit of normal for a liver abnormality; total bilirubin greater than twice the upper limit of normal for clinically important hyperbilirubinaemia; serum creatinine greater than 1.5 times baseline for a renal abnormality; and a fall in estimated glomerular filtration rate of more than 30% from baseline for meaningful renal decline. Supplement discontinuation because of an adverse event was defined as any event leading the investigator or participant to stop spirulina permanently, and a treatment-related serious adverse event as one adjudicated as possibly, probably or definitely related. MACE and non-MACE serious events are shown separately and together, as participants and as episodes, so that the total serious-event burden is visible and internally reconcilable. Full definitions are given in Supplementary Table S11.

### Statistical analysis

The analysis plan was revised during peer review and is described as a reviewer-requested reanalysis, not as prospectively prespecified. Continuous variables are reported as mean ± standard deviation and categorical variables as n (%). No single biomarker was prospectively designated as primary; all 10 biomarkers were therefore treated as exploratory. Each 3-month outcome was analyzed by ordinary least squares regression with heteroscedasticity-consistent (HC3) standard errors, adjusting for its baseline value and measured confounders: age, sex, body mass index, current smoking, hypertension, diabetes, prior myocardial infarction, prior PCI/CABG, left-ventricular ejection fraction, multivessel disease, Canadian Cardiovascular Society angina class, family support, supplement willingness, programme capacity, education, low socioeconomic status, health literacy, baseline diet quality, calendar period, baseline high-intensity statin use, and medication adherence. Benjamini-Hochberg false-discovery-rate correction was applied across the 20 comparisons of each spirulina-containing group with control. The diet-plus-spirulina vs. spirulina contrast was reported separately and does not establish synergy.

Five participants lacked paired biomarker data. The primary analysis was complete case; reasons and group counts are reported, and multiple-imputation sensitivity analysis is summarized in Supplementary Table S9. Additional sensitivity analyses excluded statin initiation/intensification and acute coronary syndrome at the index contact, adjusted for laboratory batch/plate, used extended clinical adjustment, and applied generalized-overlap weights. Two further sensitivity analyses were added in response to peer review. The first restricted every biomarker and time-to-event analysis to participants enrolled during the concurrent recruitment window of June 2020 to November 2022, so that each intervention participant was compared only with contemporaneously recruited controls. This analysis addresses the concern that drift in background care over calendar time, rather than the assigned strategy, might explain part of the between-group contrast, a recognized source of bias whenever comparison groups are not accrued concurrently (17, 18). The second restricted the supplemented groups to recipients of the earliest product batch and, separately, added a product-batch indicator to the adjusted models among supplemented participants, so that any influence of product age or batch on the 3-month outcomes could be quantified.

For the weighting sensitivity analysis, a regularized multinomial logistic model estimated generalized propensity scores from baseline clinical and treatment-selection variables. Generalized-overlap weights targeted the population with common covariate support (19). Weighted group-specific covariate values, maximum standardized mean differences, overlap plots, weight distributions and effective sample sizes are supplied. Because the maximum standardized mean difference remained 0.16 after weighting, these analyses are interpreted as sensitivity analyses rather than evidence that confounding was eliminated.

MACE-free survival was estimated by Kaplan-Meier methods. Cumulative incidence in the presence of non-cardiac death was estimated by the Aalen-Johansen method (20). Eighteen-month risk differences and percentile bootstrap 95% confidence intervals were calculated. A reviewer-requested parsimonious Cox model included group, age, sex, prior myocardial infarction, left-ventricular ejection fraction and baseline LDL cholesterol. Proportional hazards were assessed using Schoenfeld-residual tests (21), and 100 stratified bootstrap resamples assessed coefficient stability. Between-group differences in safety proportions were summarized as absolute risk differences with Newcombe hybrid-score 95% confidence intervals, without multiplicity adjustment, and are descriptive. Because the study was neither randomized nor powered for MACE, no number needed to treat was calculated and the clinical outcome was considered exploratory.

Reanalysis was performed in Python 3.13.5 with pandas 2.2.3, NumPy 2.3.5, SciPy 1.17.0, statsmodels 0.14.6, scikit-learn 1.8.0 and Matplotlib 3.10.8. The deidentified participant-level dataset and complete Python analysis script are supplied as Supplementary Data Files 1 and 2.

## Results

### Participant flow and baseline characteristics

**Figure 1:**
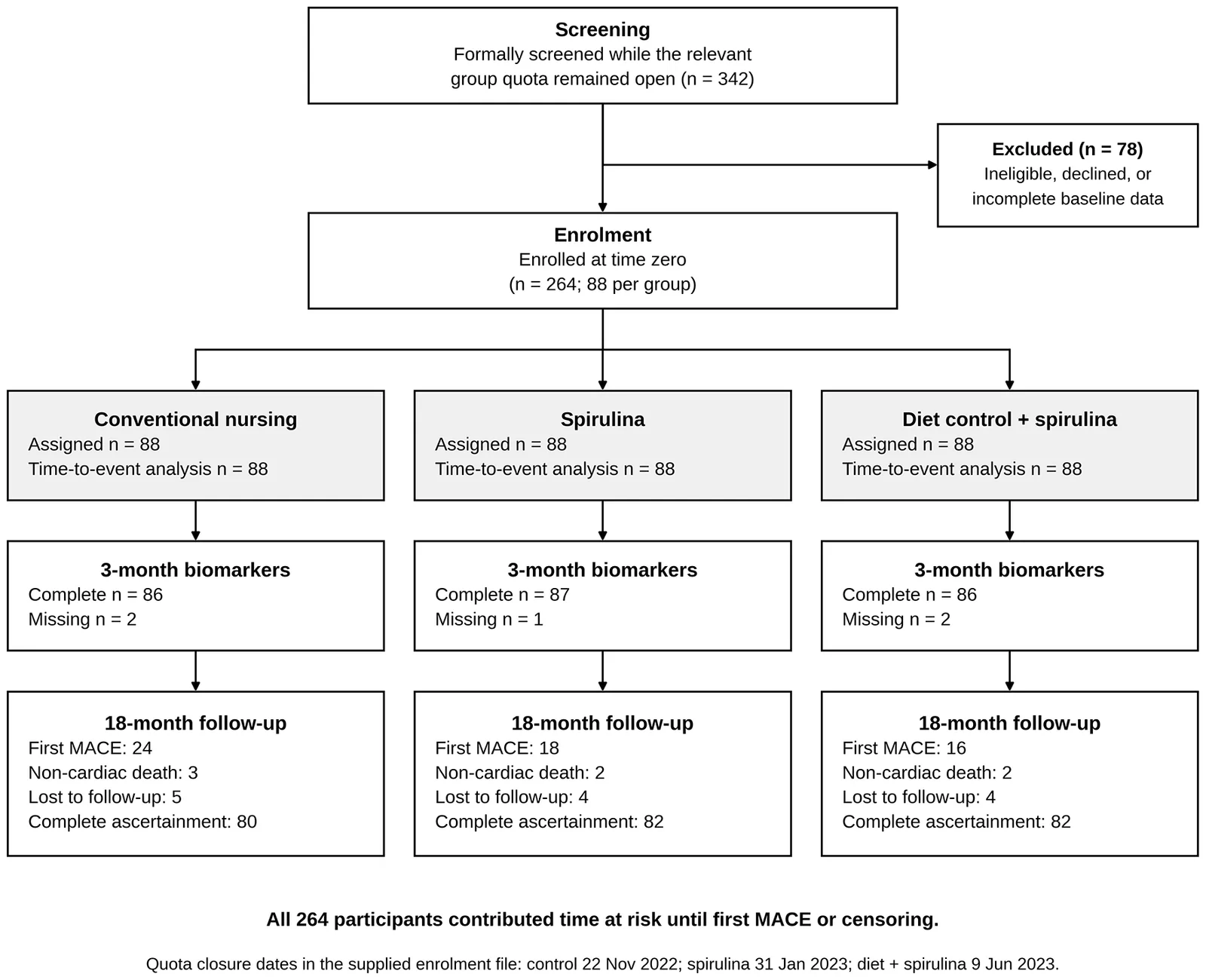
Participant flow. All 264 enrolled participants entered the time-to-event analysis at enrolment and remained under observation until first MACE or censoring. Complete 18-month endpoint ascertainment is distinct from inclusion in the survival analysis.

Of 342 formally screened patients, 78 were excluded and 264 were enrolled, 88 per group (Figure 1). All participants entered the time-to-event analysis at enrolment. Paired 3-month biomarker data were available for 86 control participants, 87 spirulina participants and 86 diet-plus-spirulina participants. Complete 18-month endpoint ascertainment was available for 80, 82, and 82 participants, respectively; participants lost to follow-up or dying from non-cardiac causes still contributed observed person-time until censoring. Enrolment was staggered because the group quotas filled at different times. All three groups recruited concurrently between June 2020 and November 2022, contributing 88 control, 81 spirulina, and 72 combined-intervention participants, whereas 7 spirulina and 16 combined-intervention participants were enrolled after the control quota closed (Supplementary Table S13; Supplementary Figure S6).

Clinical characteristics were broadly similar, but variables central to treatment selection were not balanced: adequate family support was present in 56.8, 64.8, and 73.9%; supplement willingness in 51.1, 70.5, and 76.1%; and programme capacity in 40.9, 47.7, and 68.2% (Table 1). The maximum unweighted standardized mean difference was 0.57. Generalized-overlap weighting reduced the maximum imbalance to 0.16, with effective sample sizes of 63.3, 81.8, and 63.6. Residual imbalance remained for willingness, family support, and education (Supplementary Table S3a and Figure S1).

**Table 1:** Baseline characteristics and covariate balance.

Characteristic	Control (*n* = 88)	Spirulina (*n* = 88)	Diet + spirulina (*n* = 88)	Max SMD before	Max SMD after
Age, years	66.71 ± 4.83	67.37 ± 4.79	66.89 ± 4.93	0.14	0.01
Male sex	47 (53.4)	50 (56.8)	51 (58.0)	0.09	0.05
Body mass index, kg/m^2^	24.80 ± 2.69	24.40 ± 2.49	24.20 ± 2.60	0.23	0.01
Current smoker	26 (29.5)	28 (31.8)	22 (25.0)	0.15	0.07
Hypertension	57 (64.8)	54 (61.4)	60 (68.2)	0.14	0.05
Diabetes	32 (36.4)	27 (30.7)	30 (34.1)	0.12	0.03
Previous myocardial infarction	16 (18.2)	14 (15.9)	17 (19.3)	0.09	0.04
Previous PCI/CABG	28 (31.8)	25 (28.4)	30 (34.1)	0.12	0.13
Left ventricular ejection fraction, %	54.80 ± 6.30	55.40 ± 6.19	55.20 ± 6.09	0.10	0.09
Multivessel coronary disease	42 (47.7)	39 (44.3)	40 (45.5)	0.07	0.02
CCS angina class	2.17 ± 0.86	2.11 ± 0.84	2.10 ± 0.84	0.08	0.05
Acute coronary syndrome at index contact	17 (19.3)	16 (18.2)	16 (18.2)	0.03	0.11
Adequate family support	50 (56.8)	57 (64.8)	65 (73.9)	0.36	0.16
Willingness to take supplementation	45 (51.1)	62 (70.5)	67 (76.1)	0.54	0.16
Dietary-programme capacity available	36 (40.9)	42 (47.7)	60 (68.2)	0.57	0.01
High-school education or higher	42 (47.7)	48 (54.5)	54 (61.4)	0.27	0.15
Low socioeconomic status	37 (42.0)	32 (36.4)	28 (31.8)	0.21	0.11
Health-literacy score (1–9)	5.20 ± 1.39	5.50 ± 1.40	5.80 ± 1.40	0.43	0.08
Baseline diet-quality score (1–8)	4.80 ± 1.20	5.00 ± 1.20	5.30 ± 1.20	0.42	0.06
Medication adherence, %	82.00 ± 9.00	83.99 ± 8.98	85.97 ± 7.94	0.47	0.09
High-intensity statin at baseline	39 (44.3)	42 (47.7)	40 (45.5)	0.07	0.10
Estimated GFR, mL/min/1.73 m^2^	77.00 ± 13.00	79.00 ± 12.00	78.00 ± 12.00	0.16	0.07
High-sensitivity C-reactive protein, mg/L	3.44 ± 2.66	3.54 ± 2.03	3.55 ± 1.91	0.04	0.05

Values are mean ± standard deviation or n (%). Weighted values are provided in Supplementary Table S3a. The maximum post-weighting SMD of 0.16 indicates residual imbalance.

### Three-month nutritional and cardiometabolic outcomes

**Figure 2:**
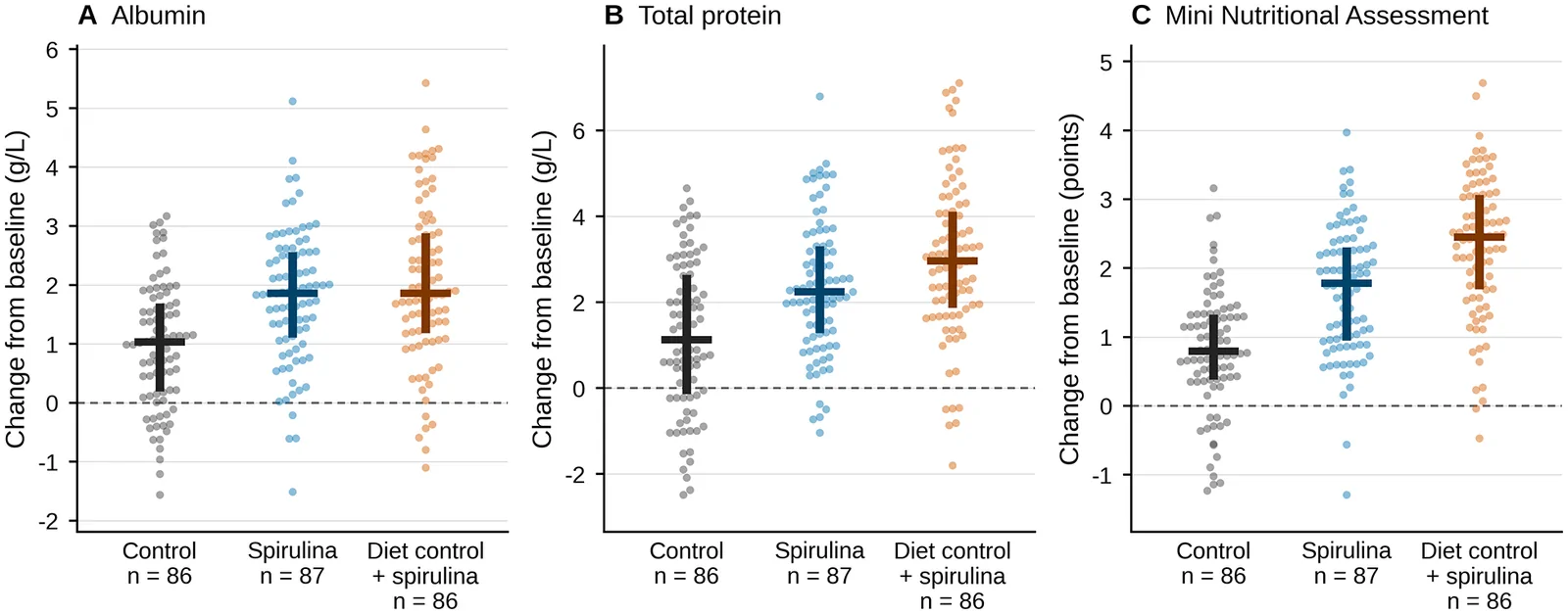
Individual 3-month changes in albumin, total protein and Mini Nutritional Assessment score. Points represent participants; thick bars represent medians and vertical bars represent interquartile ranges. **(A)** albumin, **(B)** total protein, and **(C)** Mini Nutritional Assessment score.

The newly supplied hospital dataset showed modest changes rather than the extreme shifts reported in the earlier revision. After full measured-confounder adjustment, spirulina vs. control was associated with higher albumin (+0.83 g/L, 95% CI +0.45 to +1.21), total protein (+1.23 g/L, +0.68 to +1.77) and Mini Nutritional Assessment score (+0.89 points, +0.59 to +1.19). Corresponding differences for diet plus spirulina vs. control were +1.09 g/L (+0.67 to +1.51), +1.92 g/L (+1.29 to +2.55) and +1.60 points (+1.26 to +1.95; Table 2; Figure 2).

**Table 2:** Baseline and 3-month biomarkers with adjusted between-group differences.

Outcome	Control baseline	Control 3 mo	Spirulina baseline	Spirulina 3 mo	Diet + S baseline	Diet + S 3 mo	Adjusted SP-CG (95% CI)	Adjusted DS-CG (95% CI)	Adjusted DS-SP (95% CI)
Albumin, g/L	36.30 ± 3.00	37.18 ± 3.04	36.10 ± 3.30	37.87 ± 3.51	36.20 ± 3.00	38.29 ± 3.39	+0.83 (+0.45 to +1.21)	+1.09 (+0.67 to +1.51)	+0.26 (−0.14 to +0.66)
Total protein, g/L	64.10 ± 5.40	65.21 ± 5.87	63.81 ± 5.78	66.28 ± 5.72	64.00 ± 5.30	67.01 ± 5.24	+1.23 (+0.68 to +1.77)	+1.92 (+1.29 to +2.55)	+0.69 (+0.12 to +1.27)
Mini Nutritional Assessment, points	19.00 ± 1.90	19.85 ± 2.12	18.80 ± 1.90	20.47 ± 2.25	19.10 ± 2.00	21.42 ± 2.16	+0.89 (+0.59 to +1.19)	+1.60 (+1.26 to +1.95)	+0.71 (+0.37 to +1.06)
Total cholesterol, mmol/L	5.75 ± 0.55	5.50 ± 0.61	5.72 ± 0.54	5.36 ± 0.59	5.70 ± 0.53	5.15 ± 0.56	−0.10 (−0.18 to −0.03)	−0.26 (−0.34 to −0.17)	−0.15 (−0.23 to −0.08)
Triglycerides, mmol/L	1.90 ± 0.30	1.82 ± 0.33	1.88 ± 0.29	1.73 ± 0.33	1.89 ± 0.30	1.63 ± 0.34	−0.09 (−0.14 to −0.03)	−0.20 (−0.25 to −0.15)	−0.11 (−0.16 to −0.06)
HDL cholesterol, mmol/L	1.22 ± 0.18	1.25 ± 0.19	1.20 ± 0.18	1.28 ± 0.18	1.22 ± 0.18	1.32 ± 0.20	+0.05 (+0.03 to +0.07)	+0.06 (+0.04 to +0.09)	+0.01 (−0.01 to +0.04)
LDL cholesterol, mmol/L	3.60 ± 0.38	3.27 ± 0.46	3.58 ± 0.37	3.11 ± 0.49	3.62 ± 0.39	3.03 ± 0.48	−0.12 (−0.20 to −0.04)	−0.25 (−0.33 to −0.17)	−0.13 (−0.21 to −0.05)
Homocysteine, μmol/L	14.80 ± 1.40	14.38 ± 1.44	14.60 ± 1.50	13.65 ± 1.72	14.50 ± 1.40	13.07 ± 1.56	−0.52 (−0.74 to −0.31)	−1.05 (−1.29 to −0.81)	−0.53 (−0.74 to −0.32)
Nitric oxide metabolites, μmol/L	39.00 ± 5.00	40.31 ± 5.47	38.20 ± 5.10	41.80 ± 5.87	38.80 ± 5.00	44.22 ± 5.30	+1.99 (+1.17 to +2.82)	+3.65 (+2.73 to +4.56)	+1.65 (+0.75 to +2.56)
Endothelin-1, ng/L	137.00 ± 12.00	133.23 ± 13.80	136.00 ± 13.00	130.08 ± 13.18	136.00 ± 12.00	126.22 ± 13.26	−2.18 (−3.78 to −0.58)	−5.66 (−7.48 to −3.84)	−3.48 (−5.01 to −1.96)

Paired n: control 86, spirulina 87, diet + spirulina 86. Adjusted models included baseline outcome and measured clinical and treatment-selection covariates with HC3 standard errors. Benjamini-Hochberg correction was applied to the 20 comparisons vs. control. Full p and q values are in Supplementary Table S5.

**Figure 3:**
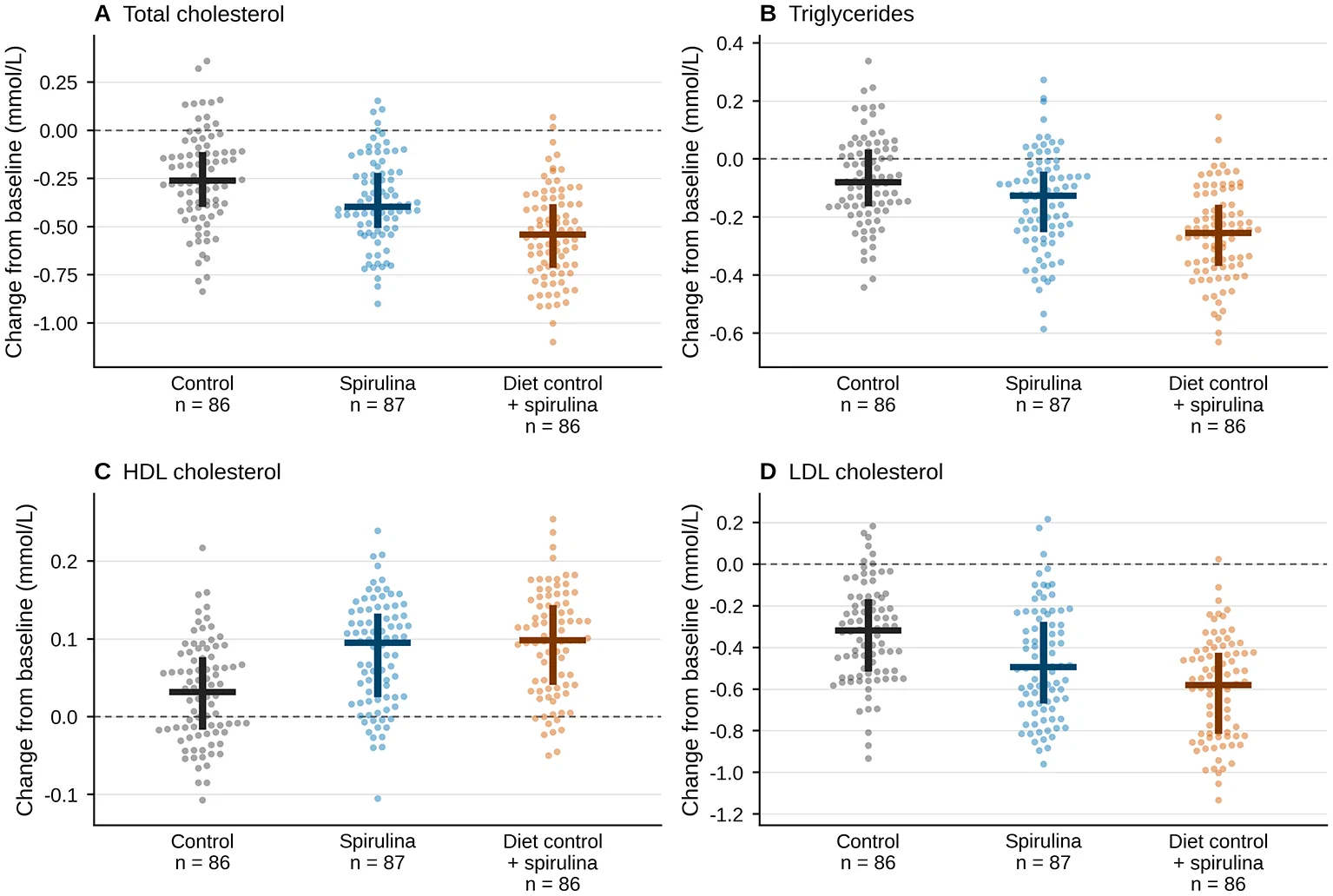
Individual 3-month changes in total cholesterol, triglycerides, HDL cholesterol, and LDL cholesterol. Points represent participants; thick bars represent medians and vertical bars represent interquartile ranges. **(A)** total cholesterol, **(B)** triglycerides, **(C)** HDL cholesterol, and **(D)** LDL cholesterol.

For lipids, adjusted spirulina-vs.-control differences were −0.10 mmol/L (−0.18 to −0.03) for total cholesterol, −0.09 mmol/L (−0.14 to −0.03) for triglycerides, +0.05 mmol/L (+0.03 to +0.07) for HDL cholesterol and −0.12 mmol/L (−0.20 to −0.04) for LDL cholesterol. Diet plus spirulina vs. control differences were−0.26, −0.20, +0.06 and −0.25 mmol/L, respectively (all false-discovery-rate *q* < 0.001 except total cholesterol and endothelin-1 in the spirulina group, which remained *q* < 0.01; Table 2; Figure 3).

**Figure 4:**
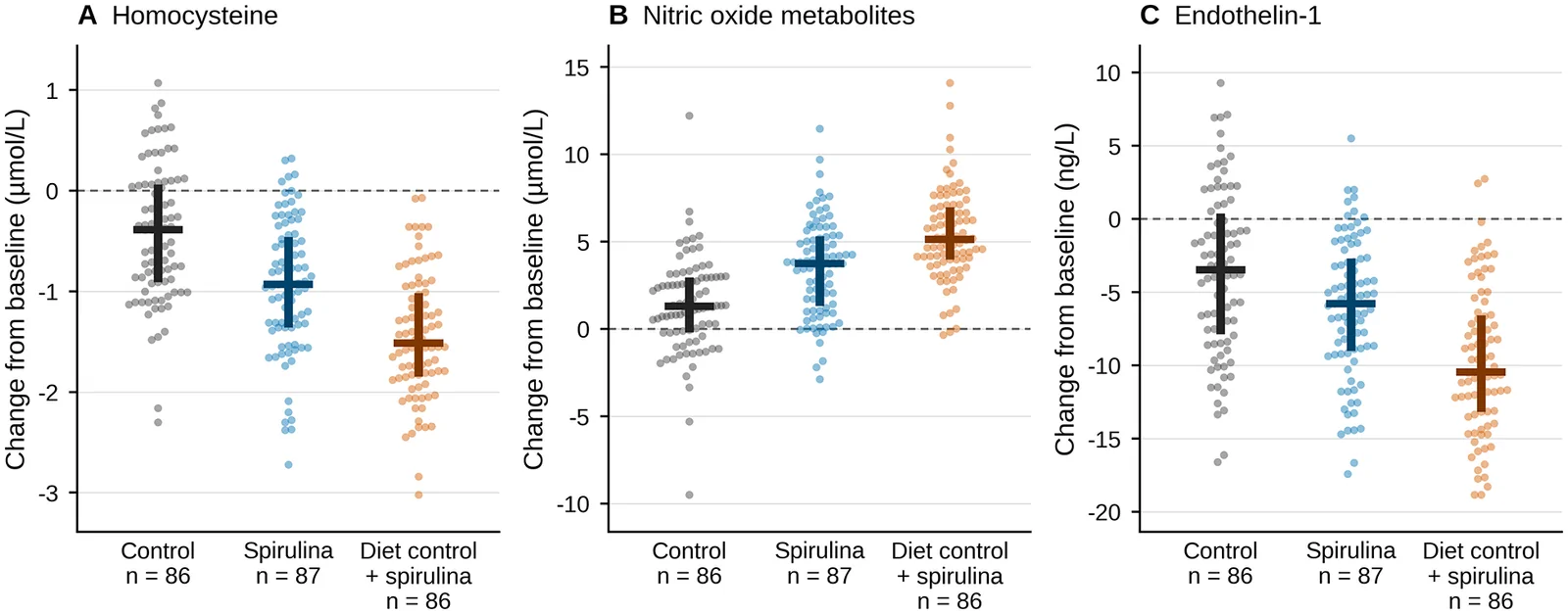
Individual 3-month changes in homocysteine, nitric-oxide metabolites and endothelin-1. Points represent participants; thick bars represent medians and vertical bars represent interquartile ranges. **(A)** homocysteine, **(B)** nitric-oxide metabolites, and **(C)** endothelin-1.

Homocysteine was lower by −0.52 μmol/L (−0.74 to −0.31) with spirulina and −1.05 μmol/L (−1.29 to −0.81) with diet plus spirulina relative to control. Nitric-oxide metabolites were higher by +1.99 μmol/L (+1.17 to +2.82) and +3.65 μmol/L (+2.73 to +4.56), whereas endothelin-1 was lower by −2.18 ng/L (−3.78 to −0.58) and −5.66 ng/L (−7.48 to −3.84; Figure 4). Results were directionally consistent in the alternative adjustment, exclusion, batch-adjusted, overlap-weighted, and multiple-imputation analyses (Supplementary Tables S5 and S9). Restricting all analyses to the 236 participants with paired data who were enrolled during the concurrent recruitment window left every estimate substantively unchanged. Adjusted spirulina-vs.-control and diet-plus-spirulina-vs.-control differences in the restricted cohort were −0.12 and −0.26 mmol/L for LDL cholesterol, −0.49 and −1.07 μmol/L for homocysteine, and +0.89 and +1.73 points for the Mini Nutritional Assessment, and all 20 contrasts with control retained significance after false-discovery-rate correction (Supplementary Table S14; Supplementary Figure S7). Estimates confined to recipients of the earliest product batch were similarly concordant, and among supplemented participants a product-batch indicator was not associated with the 3-month outcomes, with a Fisher combined *p* value of 0.156 across the ten outcomes (Supplementary Table S12).

### Adherence, continued exposure and safety

Mean 3-month spirulina adherence was 88.0% in the spirulina group and 89.0% in the combined group; 81 and 80 participants, respectively, achieved at least 80% adherence. Seventy of 88 combined-group participants achieved at least 80% dietary-checklist completion. Continued spirulina use declined after the intervention: 31, 19, and 11 spirulina-group participants and 30, 20, and 13 combined-group participants reported use at months 6, 12, and 18 (Supplementary Table S4).

Any non-serious treatment-emergent adverse event occurred in 9 control participants (10.2%), 12 spirulina participants (13.6%) and 16 combined-group participants (18.2%). Four participants discontinued supplementation because of an adverse event. Non-MACE serious adverse events occurred in 2, 1 and 2 participants and accounted for 2, 1, and 2 episodes. Counting every qualifying episode, including 2 recurrent MACE episodes in each group, the total serious-event burden was 28, 21, and 20 episodes. These episodes arose in 25 control participants (28.4%), 19 spirulina participants (21.6%) and 17 combined-group participants (19.3%); one control participant and one combined-group participant contributed to both the MACE and the non-MACE category, which is why the number of participants is smaller than the number of episodes. Corresponding episode rates were 26.9, 19.1, and 18.0 per 100 person-years. None of the serious events was adjudicated as treatment related. The full reconciliation between participants and episodes is given in Supplementary Table S15 and Supplementary Figure S8. Newcombe risk differences vs. control were +3.4 percentage points (95% CI −6.5 to +13.3) and +8.0 points (−2.5 to +18.4) for any non-serious treatment-emergent event, −1.1 points (−6.9 to +4.1) and 0.0 points (−5.9 to +5.9) for non-MACE serious events, and −6.8 points (−19.3 to +6.0) and −9.1 points (−21.4 to +3.5) for participants with any serious event including MACE; every interval included no difference (Table 3).

**Table 3:** Safety and adverse events.

Outcome	Control (*n* = 88)	Spirulina (*n* = 88)	Diet + spirulina (*n* = 88)
Gastrointestinal discomfort	2 (2.3)	5 (5.7)	6 (6.8)
Rash/allergy	0 (0.0)	1 (1.1)	1 (1.1)
Abnormal liver-function test	1 (1.1)	1 (1.1)	1 (1.1)
Renal adverse event	0 (0.0)	0 (0.0)	1 (1.1)
Other non-serious adverse event	7 (8.0)	6 (6.8)	7 (8.0)
Any non-serious treatment-emergent adverse event	9 (10.2)	12 (13.6)	16 (18.2)
Supplement discontinued because of adverse event	0 (0.0)	2 (2.3)	2 (2.3)
Non-MACE serious adverse event, participants	2 (2.3)	1 (1.1)	2 (2.3)
Participants with any serious adverse event, including MACE	25 (28.4)	19 (21.6)	17 (19.3)
First MACE episodes	24	18	16
Recurrent MACE episodes	2	2	2
Non-MACE serious-event episodes	2	1	2
Total serious-event episodes	28	21	20
Serious-event episodes per 100 person-years	26.9	19.1	18.0
Treatment-related serious adverse event	0 (0.0)	0 (0.0)	0 (0.0)

Non-serious treatment-emergent events were assessed during the 3-month intervention. Serious adverse events were assessed through 18 months. Rows expressed as n (%) count participants; rows labeled “episodes” count individual qualifying events, so that a participant contributing more than one episode, or contributing to both the MACE and the non-MACE category, is counted once among participants and once per episode among episodes. One control participant and one combined-group participant contributed to both categories. Treatment-related serious events were zero in all groups. The full reconciliation is given in Supplementary Table S15.

### Exploratory 18-month MACE

**Figure 5:**
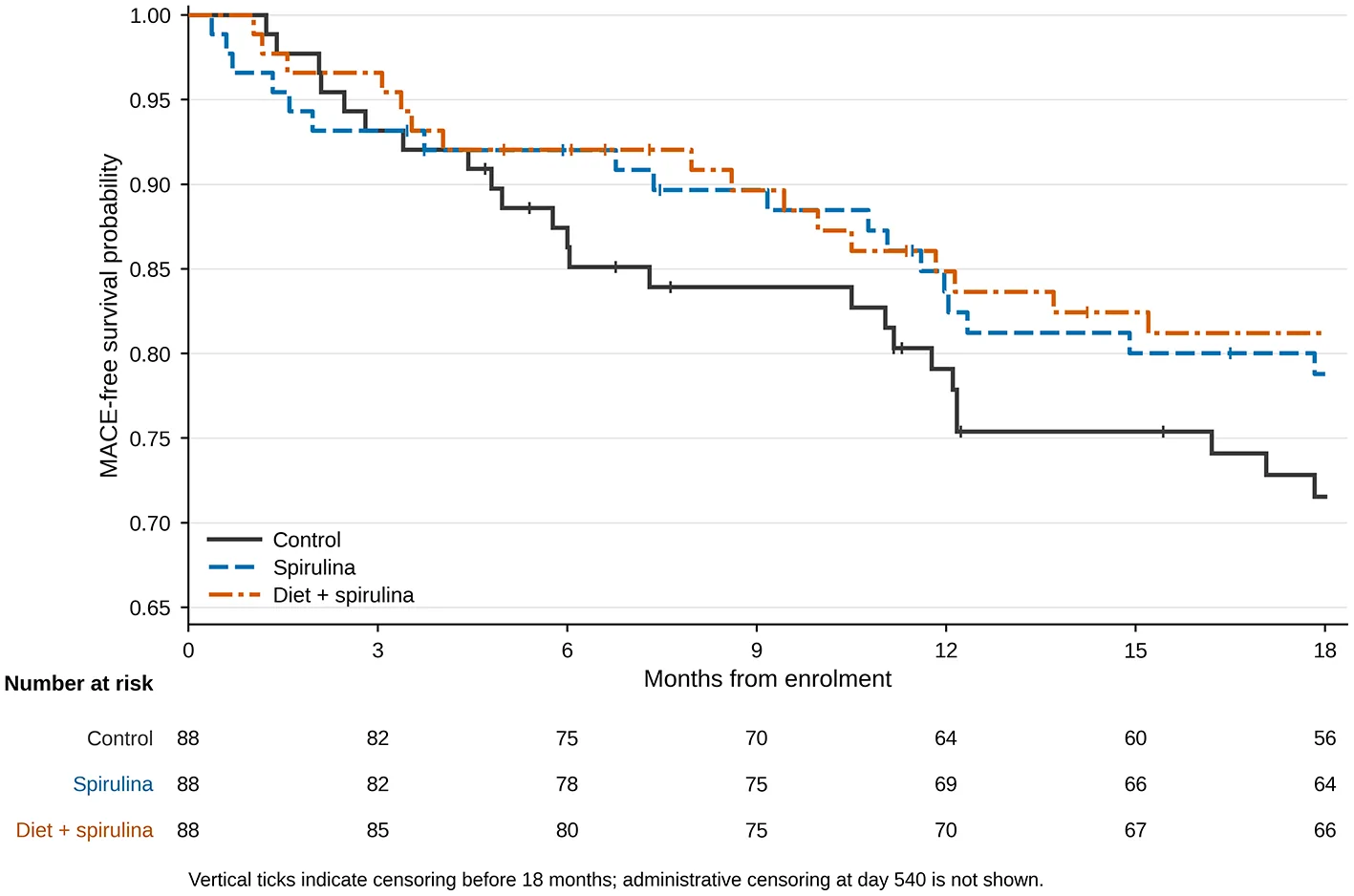
Kaplan-Meier MACE-free survival from enrolment. Vertical ticks indicate censoring before 18 months. The risk table uses exact participant-level event and censoring days.

First MACE occurred in 24 control, 18 spirulina and 16 diet-plus-spirulina participants. Exact event times were distributed throughout follow-up (Figure 5). Kaplan-Meier 18-month risks were 28.4, 21.2, and 18.8%; Aalen-Johansen cumulative incidences accounting for non-cardiac death were 28.0, 21.1, and 18.6% (Table 4; Supplementary Figure S3). Risk differences were −7.2 percentage points (95% bootstrap CI −19.4 to +6.2) for spirulina vs. control and −9.6 points (−21.5 to +2.5) for diet plus spirulina vs. control. Adjusted hazard ratios were 0.74 (95% CI 0.40–1.37; *p* = 0.341) and 0.62 (0.33–1.17; *p* = 0.142). The combined Schoenfeld-residual test gave *p* = 0.857. These estimates were compatible with both clinically relevant benefit and no difference. In the concurrent-recruitment sensitivity analysis, which comprised 88 control, 81 spirulina and 72 combined-intervention participants with 24, 17, and 13 first events, adjusted hazard ratios were 0.75 (95% CI 0.40–1.40) for spirulina and 0.63 (0.32–1.24) for diet plus spirulina, closely reproducing the full-cohort estimates (Supplementary Table S14).

**Table 4:** Exploratory 18-month major adverse cardiovascular events.

Group	First MACE, *n* (%)	MI/stroke/cardiac death	KM risk at 18 mo (%)	Aalen-Johansen CIF (%)	Risk difference vs. control, percentage points (95% CI)	Adjusted HR (95% CI)
Control	24 (27.3)	14/9/1	28.4%	28.0%	Reference	Reference
Spirulina	18 (20.5)	9/8/1	21.2%	21.1%	−7.2 (−19.4 to +6.2)	0.74 (0.40–1.37); *p* = 0.341
Diet + spirulina	16 (18.2)	7/5/4	18.8%	18.6%	−9.6 (−21.5 to +2.5)	0.62 (0.33–1.17); *p* = 0.142

Risk-difference CIs are percentile bootstrap intervals. The Cox model adjusted for age, sex, prior myocardial infarction, left-ventricular ejection fraction and baseline LDL cholesterol. Non-cardiac death was treated as a competing event for cumulative incidence.

## Discussion

This reanalysis supports a more limited and defensible interpretation than the earlier revision. In older adults with coronary artery disease, the two spirulina-containing strategies were associated with small favorable differences across nutritional, lipid, homocysteine and endothelial biomarkers at 3 months. Differences were generally larger when spirulina was delivered within structured nursing-led dietary management. However, MACE estimates were imprecise and did not establish a clinical benefit.

The revised effect sizes are more consistent with existing human evidence than the earlier values. Randomized trials and meta-analyses suggest that spirulina can produce modest lipid changes, but findings are heterogeneous across doses, formulations and populations (8–11). The present study adds a clinically pragmatic comparison in older patients with coronary disease, while its nonrandomized assignment makes it less informative about causality than a randomized trial. Measured variables that influenced assignment—willingness, family support, programme availability, health literacy and diet quality—were included in the outcome models. Even so, overlap weighting did not achieve complete balance, and unmeasured healthy-adherer behavior remains a plausible explanation for part of the observed differences.

The homocysteine result should not be interpreted as evidence that spirulina supplied bioavailable vitamin B12. Analytical studies show that pseudovitamin B12 predominates in spirulina products and is not an appropriate substitute for active cobalamin in humans (6, 7). The supplied certificate similarly describes predominantly pseudovitamin B12, and neither serum B12, folate, methylmalonic acid, nor holotranscobalamin was measured. The observed −0.52 to −1.05 μmol/L adjusted differences may reflect broader dietary change, regression to the mean, medication adherence, renal or inflammatory factors, or residual confounding. Moreover, B-vitamin homocysteine lowering has not consistently reduced cardiovascular events (4, 5), so the biomarker should not be used as a surrogate for MACE benefit.

The larger differences for the combined group are compatible with an incremental association of the structured programme, but the study has no dietary-management-only group. It therefore cannot isolate a dietary effect or demonstrate synergy. Checklist completion records delivery of the nursing process, not achievement of nutrient targets. Quantitative repeated dietary recalls, food records or intake biomarkers were not collected.

The MACE analysis was rebuilt using exact event and censoring days, a correct risk table and non-cardiac death as a competing event. The directions of the estimates favored both spirulina-containing groups, but all confidence intervals included no difference. Continued spirulina exposure after month 3 also declined substantially. A causal interpretation of an 18-month effect from a 3-month nonrandomized intervention would therefore be particularly fragile. The appropriate conclusion is that the MACE findings are hypothesis generating.

Three specific aspects of study conduct warrant explicit comment because they bear directly on how the estimates should be read. The first is product accountability. Three sequential batches were dispensed over a 3-year enrolment window, the pharmacy record documents lot number, expiry date and dispensing period for each, and reconciliation of dispensing dates against expiry dates shows that no participant received or consumed product beyond its labeled shelf life. Adherence and adverse-event frequencies were closely similar across batches, and analyses restricted to the earliest batch reproduced the main estimates, so product age is an implausible explanation for the differences reported here. The second is the staggered recruitment that followed from closing each group quota when it filled, which left 23 intervention participants without contemporaneously recruited controls. Comparison groups accrued at different calendar times can yield biased effect estimates when background care or case mix drifts, and this concern is well described in the literature on non-concurrent controls (17, 18). We therefore repeated every analysis within the concurrent window; the estimates were essentially unchanged, which argues against secular drift as a material source of bias, although a restricted analysis of this size cannot exclude it. The third is the accounting of serious events. The earlier presentation combined participant counts and episode counts in a single row, which made the totals appear irreconcilable. Serious events are now reported in both metrics throughout, since a participant may experience more than one qualifying episode and may contribute to more than one category (16).

Strengths include prospective enrolment, fixed assignment at time zero, participant-level source data, exact event and censoring times, explicit treatment-selection variables, synchronized tables and figures, multiplicity control, competing-risk estimation, and provision of the deidentified dataset and analysis code. The analysis also separates complete endpoint ascertainment from inclusion in the time-to-event population.

Several limitations are decisive. First, the design was nonrandomized, and residual confounding remains despite extensive adjustment. Second, quota-limited recruitment generated equal group sizes but closed the group quotas on different calendar dates, so 23 intervention participants lacked contemporaneously recruited controls, and the available screening log does not enumerate potentially eligible patients encountered after a quota closed; the concurrent-window sensitivity analysis was reassuring but is necessarily smaller and cannot exclude residual calendar-time confounding. Third, the generalized-overlap sensitivity analysis retained a maximum standardized mean difference of 0.16. Fourth, the biomarker hierarchy and revised models were selected during peer review rather than prospectively specified. Fifth, there was no dietary-management-only arm and no objective quantitative dietary assessment. Sixth, five paired biomarker assessments were missing, although complete-case and multiple-imputation results were similar. Seventh, statin initiation or intensification and recovery after an index cardiac contact may contribute to short-term changes despite sensitivity analyses. Eighth, no direct B-vitamin-status measurements were available. Ninth, the study was small for hard cardiovascular outcomes, and post-month-3 spirulina exposure was neither sustained nor randomized. Tenth, the study was not registered in a public clinical-trials registry before enrolment, so protection against selective outcome reporting rests on the supplied source records rather than on a prospectively posted protocol. Finally, a prospectively registered, adequately powered randomized factorial trial is required to distinguish spirulina, structured dietary management and their interaction.

## Conclusion

Spirulina with routine guidance and spirulina within a nursing-led dietary programme were associated with modestly more favorable nutritional, lipid, homocysteine and endothelial biomarker profiles at 3 months than conventional nursing care. The combined programme showed larger adjusted differences for several outcomes, but the design cannot establish an independent dietary effect or synergy. Eighteen-month MACE estimates were directionally favorable but statistically inconclusive. These findings should be treated as exploratory and should inform, rather than substitute for, a prospectively registered randomized trial.

## Data Availability

The original contributions presented in the study are included in the article/Supplementary material, further inquiries can be directed to the corresponding author.
