# Supplementary material for: Three-month biomarker changes after spirulina with or without nursing-led dietary management in older adults with coronary artery disease: a prospective nonrandomized comparative study
Source: Front Med (Lausanne). 2026 Aug 14;13:1910826. doi: 10.3389/fmed.2026.1910826 (PMC13522770; doi:10.3389/fmed.2026.1910826)
Supplement: Supplementary file 1 [file Data_Sheet_1.docx]

**Supplementary material**

This file contains supplementary tables only. The deidentified dataset, data dictionary, analysis code and machine-readable outputs are supplied separately.

**Supplementary Table S1. Structured nursing-led dietary-management protocol.**

| **Component** | **Recorded protocol** |
| --- | --- |
| Initial assessment | Habitual diet, appetite, dentition/swallowing, food access, family support, comorbidities and medications reviewed at enrolment. |
| Sodium | Target <5 g salt/day; avoid high-sodium processed foods; family taught label reading and household measurement. |
| Fat and cholesterol | Fat target <30% of energy; cholesterol <300 mg/day; replace animal fats with unsaturated oils; limit offal, fatty meat and egg yolk. |
| Protein | Target 15-20% of energy, emphasizing lean and plant protein while considering renal function. |
| Carbohydrate and fibre | Target 50-65% of energy, favouring whole grains, vegetables, fruit and legumes. |
| Delivery | Individualized menu, nurse counselling, family involvement, checklist documentation and scheduled review during the first 3 months. |
| Maintenance | Dietary targets reinforced at months 6, 12 and 18; no new individualized menu was issued after month 3. |

**Supplementary Table S2. Spirulina product information transcribed from the product label, purchase record and manufacturer’s signed certificate of analysis.**

| **Field** | **Information** |
| --- | --- |
| Dose form and regimen | One 700-mg Arthrospira biomass tablet after each main meal; 2.1 g/day for 3 months. |
| Fortification | Supplied certificate records no added cyanocobalamin or folic acid. |
| Total corrinoids | 1.18 µg/g by LC-MS/MS (supplied certificate). |
| Pseudovitamin B12 | 1.07 µg/g (supplied certificate). |
| Authentic cobalamin | 0.04 µg/g (supplied certificate). |
| Commercial name | Spirulina 700. |
| Manufacturer | Nutraceutical Manufacturing Co., China. |
| Lot/batch number | Batch A, NIS21090450-SRL-09; Batch B, SIM-SP-B12-20220915-02; Batch C, SIM-SP-B12-20230220-01. Full batch accountability is given in Supplementary Table S12. |
| Manufacturing date | Batch A, January 2020; Batch B, September 2022; Batch C, February 2023. |
| Expiry date | Batch A, 31 December 2022; Batch B, 30 June 2024; Batch C, 31 December 2024. No participant received or consumed product after the expiry date of the batch dispensed to them. |
| Identity confirmation | Appearance, light microscopy and manufacturer identity testing. |
| Corrinoid method | LC-MS/MS. |
| Heavy metals | Reported below the applicable food-supplement limits. |
| Microbiological testing | Total aerobic count, yeasts and moulds, coliforms, Salmonella species and Staphylococcus aureus tested. |
| Certificate status | Signed certificate of analysis matched to the stated product lot. |
| Interpretive note | Product identifiers, dates and analytical results are transcribed from the manufacturer’s signed certificate of analysis, the product label and the study-site purchase record. They are source-document transcriptions and were not independently re-analysed by the investigators. |

**Supplementary Table S3a. Full unweighted and generalized-overlap-weighted covariate balance.**

| **Covariate** | **CG unweighted** | **SP unweighted** | **DS unweighted** | **CG weighted** | **SP weighted** | **DS weighted** | **Max SMD before** | **Max SMD after** | **Missing n** |
| --- | --- | --- | --- | --- | --- | --- | --- | --- | --- |
| age_years | 66.708 | 67.368 | 66.889 | 66.921 | 66.922 | 66.884 | 0.137 | 0.008 | 0 |
| male | 0.534 | 0.568 | 0.580 | 0.561 | 0.561 | 0.535 | 0.091 | 0.053 | 0 |
| BMI_kg_m2 | 24.801 | 24.402 | 24.199 | 24.493 | 24.478 | 24.462 | 0.228 | 0.012 | 0 |
| current_smoker | 0.295 | 0.318 | 0.250 | 0.276 | 0.294 | 0.261 | 0.151 | 0.074 | 0 |
| hypertension | 0.648 | 0.614 | 0.682 | 0.646 | 0.623 | 0.636 | 0.142 | 0.048 | 0 |
| diabetes | 0.364 | 0.307 | 0.341 | 0.351 | 0.339 | 0.338 | 0.120 | 0.027 | 0 |
| dyslipidaemia | 0.727 | 0.750 | 0.784 | 0.752 | 0.732 | 0.788 | 0.132 | 0.131 | 0 |
| prior_MI | 0.182 | 0.159 | 0.193 | 0.139 | 0.155 | 0.150 | 0.089 | 0.043 | 0 |
| prior_PCI_CABG | 0.318 | 0.284 | 0.341 | 0.363 | 0.304 | 0.334 | 0.122 | 0.126 | 0 |
| CAD_duration_years | 2.420 | 2.380 | 2.400 | 2.400 | 2.401 | 2.445 | 0.070 | 0.076 | 0 |
| LVEF_pct | 54.800 | 55.400 | 55.198 | 55.609 | 55.080 | 55.329 | 0.096 | 0.085 | 0 |
| multivessel_CAD | 0.477 | 0.443 | 0.455 | 0.468 | 0.459 | 0.462 | 0.068 | 0.018 | 0 |
| CCS_angina_class | 2.170 | 2.114 | 2.102 | 2.125 | 2.095 | 2.134 | 0.080 | 0.046 | 0 |
| index_contact_within_7d | 0.580 | 0.602 | 0.591 | 0.564 | 0.602 | 0.580 | 0.046 | 0.077 | 0 |
| ACS_at_index | 0.193 | 0.182 | 0.182 | 0.167 | 0.182 | 0.142 | 0.029 | 0.107 | 0 |
| family_support_adequate | 0.568 | 0.648 | 0.739 | 0.634 | 0.662 | 0.707 | 0.362 | 0.156 | 0 |
| supplement_willingness | 0.511 | 0.705 | 0.761 | 0.634 | 0.671 | 0.709 | 0.535 | 0.160 | 0 |
| programme_capacity_available | 0.409 | 0.477 | 0.682 | 0.513 | 0.513 | 0.510 | 0.566 | 0.007 | 0 |
| education_high_school_plus | 0.477 | 0.545 | 0.614 | 0.561 | 0.533 | 0.607 | 0.275 | 0.151 | 0 |
| low_SES | 0.420 | 0.364 | 0.318 | 0.334 | 0.371 | 0.321 | 0.212 | 0.106 | 0 |
| health_literacy_score_1_9 | 5.199 | 5.500 | 5.800 | 5.462 | 5.475 | 5.578 | 0.431 | 0.084 | 0 |
| baseline_diet_quality_score_1_8 | 4.799 | 5.000 | 5.300 | 5.019 | 5.073 | 5.087 | 0.418 | 0.061 | 0 |
| high_ward_workload | 0.443 | 0.398 | 0.352 | 0.390 | 0.380 | 0.377 | 0.185 | 0.027 | 0 |
| high_intensity_statin_baseline | 0.443 | 0.477 | 0.455 | 0.474 | 0.466 | 0.517 | 0.068 | 0.102 | 0 |
| medication_adherence_pct | 82.001 | 83.989 | 85.967 | 83.204 | 83.798 | 83.907 | 0.467 | 0.085 | 0 |
| antiplatelet | 0.909 | 0.886 | 0.909 | 0.896 | 0.898 | 0.890 | 0.075 | 0.027 | 0 |
| ACEI_ARB | 0.602 | 0.568 | 0.625 | 0.609 | 0.594 | 0.628 | 0.115 | 0.070 | 0 |
| beta_blocker | 0.670 | 0.693 | 0.716 | 0.698 | 0.704 | 0.699 | 0.098 | 0.012 | 0 |
| eGFR_mL_min_1_73m2 | 76.998 | 79.001 | 77.999 | 77.498 | 78.331 | 77.823 | 0.160 | 0.069 | 0 |
| hsCRP_mg_L | 3.441 | 3.540 | 3.545 | 3.401 | 3.522 | 3.513 | 0.045 | 0.051 | 0 |
| LDL_baseline_mmol_L | 3.600 | 3.580 | 3.620 | 3.583 | 3.623 | 3.586 | 0.105 | 0.106 | 0 |
| Hcy_baseline_umol_L | 14.800 | 14.601 | 14.500 | 14.687 | 14.672 | 14.609 | 0.214 | 0.056 | 0 |
| calendar_period | 2.023 | 1.989 | 2.000 | 2.037 | 1.997 | 1.920 | 0.042 | 0.145 | 0 |

Binary variables are proportions; continuous variables are means. The largest post-weighting SMD was 0.160, so weighted analyses do not eliminate measured confounding.

**Supplementary Table S3b. Generalized-overlap-weight diagnostics.**

| **Group** | **n** | **Minimum** | **1st percentile** | **Median** | **Mean** | **99th percentile** | **Maximum** | **Effective sample size** |
| --- | --- | --- | --- | --- | --- | --- | --- | --- |
| Control | 88 | 0.040 | 0.079 | 0.845 | 1.000 | 2.525 | 2.789 | 63.3 |
| Spirulina | 88 | 0.509 | 0.521 | 0.966 | 1.000 | 1.839 | 2.044 | 81.8 |
| Diet + spirulina | 88 | 0.187 | 0.190 | 0.869 | 1.000 | 2.480 | 2.742 | 63.6 |

**Supplementary Table S4. Adherence and post-month-3 spirulina continuation.**

| **Group** | **Mean spirulina adherence** | **Spirulina adherence ≥80%, n** | **Mean diet-checklist completion** | **Diet checklist ≥80%, n** | **Continued at month 6** | **Month 12** | **Month 18** |
| --- | --- | --- | --- | --- | --- | --- | --- |
| Control | - | 0 | - | 0 | 0 | 0 | 0 |
| Spirulina | 88.0% | 81 | - | 0 | 31 | 19 | 11 |
| Diet + spirulina | 89.0% | 80 | 86.2% | 70 | 30 | 20 | 13 |

**Supplementary Table S5. Sensitivity analyses for selected key outcomes.**

| **Outcome** | **Analysis** | **Contrast** | **Adjusted difference (95% CI)** | **p** | **n** |
| --- | --- | --- | --- | --- | --- |
| MNA | Minimal adjustment | SP-CG | +0.86 (+0.58 to +1.13) | <0.0001 | 259 |
| MNA | Minimal adjustment | DS-CG | +1.50 (+1.20 to +1.79) | <0.0001 | 259 |
| MNA | Full measured-confounder adjustment | SP-CG | +0.89 (+0.59 to +1.19) | <0.0001 | 259 |
| MNA | Full measured-confounder adjustment | DS-CG | +1.60 (+1.26 to +1.95) | <0.0001 | 259 |
| MNA | Extended clinical adjustment | SP-CG | +0.89 (+0.59 to +1.19) | <0.0001 | 259 |
| MNA | Extended clinical adjustment | DS-CG | +1.58 (+1.24 to +1.93) | <0.0001 | 259 |
| MNA | Exclude statin initiation/intensification | SP-CG | +1.05 (+0.66 to +1.44) | <0.0001 | 171 |
| MNA | Exclude statin initiation/intensification | DS-CG | +1.74 (+1.32 to +2.15) | <0.0001 | 171 |
| MNA | Exclude acute coronary syndrome at index | SP-CG | +0.87 (+0.52 to +1.22) | <0.0001 | 211 |
| MNA | Exclude acute coronary syndrome at index | DS-CG | +1.62 (+1.25 to +2.00) | <0.0001 | 211 |
| MNA | Laboratory batch/plate adjusted | SP-CG | +0.85 (+0.47 to +1.24) | <0.0001 | 259 |
| MNA | Laboratory batch/plate adjusted | DS-CG | +1.67 (+1.31 to +2.03) | <0.0001 | 259 |
| MNA | Generalized-overlap weighted | SP-CG | +0.93 (+0.64 to +1.22) | <0.0001 | 259 |
| MNA | Generalized-overlap weighted | DS-CG | +1.49 (+1.14 to +1.83) | <0.0001 | 259 |
| LDL | Minimal adjustment | SP-CG | -0.13 (-0.21 to -0.06) | 0.0005 | 259 |
| LDL | Minimal adjustment | DS-CG | -0.27 (-0.34 to -0.20) | <0.0001 | 259 |
| LDL | Full measured-confounder adjustment | SP-CG | -0.12 (-0.20 to -0.04) | 0.0030 | 259 |
| LDL | Full measured-confounder adjustment | DS-CG | -0.25 (-0.33 to -0.17) | <0.0001 | 259 |
| LDL | Extended clinical adjustment | SP-CG | -0.12 (-0.18 to -0.06) | 0.0001 | 259 |
| LDL | Extended clinical adjustment | DS-CG | -0.25 (-0.32 to -0.19) | <0.0001 | 259 |
| LDL | Exclude statin initiation/intensification | SP-CG | -0.10 (-0.18 to -0.01) | 0.0229 | 171 |
| LDL | Exclude statin initiation/intensification | DS-CG | -0.24 (-0.32 to -0.15) | <0.0001 | 171 |
| LDL | Exclude acute coronary syndrome at index | SP-CG | -0.15 (-0.24 to -0.06) | 0.0006 | 211 |
| LDL | Exclude acute coronary syndrome at index | DS-CG | -0.27 (-0.36 to -0.18) | <0.0001 | 211 |
| LDL | Laboratory batch/plate adjusted | SP-CG | -0.13 (-0.23 to -0.03) | 0.0082 | 259 |
| LDL | Laboratory batch/plate adjusted | DS-CG | -0.24 (-0.33 to -0.15) | <0.0001 | 259 |
| LDL | Generalized-overlap weighted | SP-CG | -0.14 (-0.22 to -0.06) | 0.0003 | 259 |
| LDL | Generalized-overlap weighted | DS-CG | -0.25 (-0.33 to -0.17) | <0.0001 | 259 |
| Hcy | Minimal adjustment | SP-CG | -0.53 (-0.73 to -0.33) | <0.0001 | 259 |
| Hcy | Minimal adjustment | DS-CG | -1.04 (-1.24 to -0.84) | <0.0001 | 259 |
| Hcy | Full measured-confounder adjustment | SP-CG | -0.52 (-0.74 to -0.31) | <0.0001 | 259 |
| Hcy | Full measured-confounder adjustment | DS-CG | -1.05 (-1.29 to -0.81) | <0.0001 | 259 |
| Hcy | Extended clinical adjustment | SP-CG | -0.52 (-0.74 to -0.30) | <0.0001 | 259 |
| Hcy | Extended clinical adjustment | DS-CG | -1.04 (-1.28 to -0.80) | <0.0001 | 259 |
| Hcy | Exclude statin initiation/intensification | SP-CG | -0.52 (-0.79 to -0.25) | 0.0001 | 171 |
| Hcy | Exclude statin initiation/intensification | DS-CG | -1.16 (-1.44 to -0.87) | <0.0001 | 171 |
| Hcy | Exclude acute coronary syndrome at index | SP-CG | -0.61 (-0.86 to -0.36) | <0.0001 | 211 |
| Hcy | Exclude acute coronary syndrome at index | DS-CG | -1.13 (-1.40 to -0.87) | <0.0001 | 211 |
| Hcy | Laboratory batch/plate adjusted | SP-CG | -0.57 (-0.82 to -0.31) | <0.0001 | 259 |
| Hcy | Laboratory batch/plate adjusted | DS-CG | -1.03 (-1.28 to -0.77) | <0.0001 | 259 |
| Hcy | Generalized-overlap weighted | SP-CG | -0.53 (-0.75 to -0.30) | <0.0001 | 259 |
| Hcy | Generalized-overlap weighted | DS-CG | -1.05 (-1.29 to -0.82) | <0.0001 | 259 |
| ET1 | Minimal adjustment | SP-CG | -2.52 (-4.11 to -0.92) | 0.0019 | 259 |
| ET1 | Minimal adjustment | DS-CG | -6.16 (-7.78 to -4.54) | <0.0001 | 259 |
| ET1 | Full measured-confounder adjustment | SP-CG | -2.18 (-3.78 to -0.58) | 0.0075 | 259 |
| ET1 | Full measured-confounder adjustment | DS-CG | -5.66 (-7.48 to -3.84) | <0.0001 | 259 |
| ET1 | Extended clinical adjustment | SP-CG | -2.20 (-3.87 to -0.53) | 0.0099 | 259 |
| ET1 | Extended clinical adjustment | DS-CG | -5.65 (-7.55 to -3.75) | <0.0001 | 259 |
| ET1 | Exclude statin initiation/intensification | SP-CG | -0.75 (-2.86 to +1.37) | 0.4887 | 171 |
| ET1 | Exclude statin initiation/intensification | DS-CG | -5.18 (-7.56 to -2.80) | <0.0001 | 171 |
| ET1 | Exclude acute coronary syndrome at index | SP-CG | -2.76 (-4.53 to -0.98) | 0.0023 | 211 |
| ET1 | Exclude acute coronary syndrome at index | DS-CG | -5.91 (-8.01 to -3.80) | <0.0001 | 211 |
| ET1 | Laboratory batch/plate adjusted | SP-CG | -3.77 (-7.31 to -0.23) | 0.0369 | 259 |
| ET1 | Laboratory batch/plate adjusted | DS-CG | -6.39 (-12.60 to -0.18) | 0.0439 | 259 |
| ET1 | Generalized-overlap weighted | SP-CG | -2.29 (-4.13 to -0.44) | 0.0153 | 259 |
| ET1 | Generalized-overlap weighted | DS-CG | -6.04 (-8.12 to -3.96) | <0.0001 | 259 |

The complete 210-row sensitivity-output table is supplied as Analysis/TableS5_Sensitivity.csv.

**Supplementary Table S6. Cox-model stability and proportional-hazards diagnostics.**

| **Contrast** | **Cox HR (95% CI)** | **p** | **Bootstrap median HR (2.5th to 97.5th percentile)** | **Successful resamples** |
| --- | --- | --- | --- | --- |
| Spirulina vs control | 0.742 (0.402 to 1.371) | 0.341 | 0.722 (0.469 to 1.426) | 100 |
| Diet + spirulina vs control | 0.621 (0.328 to 1.174) | 0.142 | 0.596 (0.318 to 1.093) | 100 |

| **Term** | **Schoenfeld residual-time correlation** | **p** |
| --- | --- | --- |
| SP | -0.121 | 0.365 |
| DS | 0.008 | 0.953 |
| age_years | 0.154 | 0.248 |
| male | -0.046 | 0.731 |
| prior_MI | 0.074 | 0.580 |
| LVEF_pct | -0.114 | 0.396 |
| LDL_baseline_mmol_L | -0.008 | 0.951 |

Combined proportional-hazards test p = 0.857.

**Supplementary Table S7. Dietary-process documentation in the diet-plus-spirulina group.**

| **Process item** | **Documented, n** | **Percentage** |
| --- | --- | --- |
| Salt target documented | 78 | 88.6% |
| Cholesterol target documented | 74 | 84.1% |
| Fat target documented | 76 | 86.4% |
| Protein target documented | 72 | 81.8% |
| Carbohydrate target documented | 75 | 85.2% |
| Fibre/produce target documented | 80 | 90.9% |

**Supplementary Table S8. Clinical setting, medication changes and enrolment period.**

| **Group** | **Index contact ≤7 d** | **ACS at index** | **Days index to baseline, median (IQR)** | **Statin new/intensified** | **High-intensity statin baseline** | **High-intensity statin 3 mo** | **First enrolment** | **Quota closure** |
| --- | --- | --- | --- | --- | --- | --- | --- | --- |
| Control | 51 | 17 | 7 (4-14) | 29 | 39 | 56 | 2020-06-30 | 2022-11-22 |
| Spirulina | 53 | 16 | 6 (3-17) | 31 | 42 | 57 | 2020-06-07 | 2023-01-31 |
| Diet + spirulina | 52 | 16 | 6 (2-19) | 30 | 40 | 52 | 2020-06-20 | 2023-06-09 |

**Supplementary Table S9. Missing paired biomarker data and multiple-imputation sensitivity analysis.**

| **Group** | **Complete paired data** | **Missing** | **Withdrew consent** | **Relocated** |
| --- | --- | --- | --- | --- |
| Control | 86 | 2 | 1 | 1 |
| Spirulina | 87 | 1 | 1 | 0 |
| Diet + spirulina | 86 | 2 | 2 | 0 |

| **Outcome** | **Contrast** | **Multiple-imputation adjusted difference (95% CI)** |
| --- | --- | --- |
| Albumin | SP-CG | +0.82 (+0.48 to +1.17) |
| Albumin | DS-CG | +1.03 (+0.69 to +1.38) |
| MNA | SP-CG | +0.85 (+0.57 to +1.14) |
| MNA | DS-CG | +1.49 (+1.20 to +1.78) |
| LDL cholesterol | SP-CG | -0.12 (-0.17 to -0.07) |
| LDL cholesterol | DS-CG | -0.26 (-0.31 to -0.20) |
| Homocysteine | SP-CG | -0.53 (-0.72 to -0.33) |
| Homocysteine | DS-CG | -1.04 (-1.23 to -0.84) |

Multiple-imputation values were transcribed from the supplied Table_MI_Sensitivity.xlsx worksheet (20 imputations). The primary reproducibility script uses complete-case analyses and does not regenerate these imputed datasets.

**Supplementary Table S10. Laboratory methods and quality-control information transcribed from the supplied laboratory-validation worksheet.**

| **Analyte** | **Method** | **Analytical range** | **Quality control / action** |
| --- | --- | --- | --- |
| Albumin | Bromocresol green | 20-60 g/L | Within-run CV 1.7%; between-run CV 2.5% |
| Total protein | Biuret | 30-100 g/L | Within-run CV 1.5%; between-run CV 2.3% |
| Total cholesterol | CHOD-PAP | 0.5-15 mmol/L | Within-run CV 1.6%; between-run CV 2.4% |
| Triglycerides | GPO-PAP | 0.1-10 mmol/L | Within-run CV 1.8%; between-run CV 2.7% |
| HDL/LDL cholesterol | Direct homogeneous assays | Worksheet-specific analytical ranges | Within-run CV ≤2.2%; between-run CV ≤3.0% |
| Homocysteine | Enzymatic cycling | 3-50 µmol/L | Within-run CV 2.4%; between-run CV 3.2% |
| Nitric-oxide metabolites | Nitrate reductase/Griess | Worksheet-specific range | Within-run CV 4.1%; between-run CV 5.6% |
| Endothelin-1 | Sandwich ELISA | Worksheet-specific range | Within-run CV 4.6%; between-run CV 6.2% |
| Instrument and kit identifiers | Roche cobas c 311 clinical-chemistry analyser; Thermo Scientific Multiskan FC microplate reader | Reagent, kit and instrument catalogue numbers are listed in Supplementary Table S10a | Manufacturer-assigned calibrators; two levels of internal quality control analysed daily and accepted only within predefined control limits |

**Supplementary Table S10a. Instrument, reagent and kit identifiers for the laboratory outcome measurements.**

| **Item** | **Manufacturer, product and catalogue/material number** |
| --- | --- |
| Chemistry analyser | Roche cobas c 311 automated clinical-chemistry analyser; Roche material no. 04826876001. |
| Albumin reagent | Roche ALB2 Albumin Gen.2 (bromocresol green); material no. 03183688122. |
| Total-protein reagent | Roche TP2 Total Protein Gen.2 (biuret); material no. 03183734190. |
| Total-cholesterol reagent | Roche CHOL2 Cholesterol Gen.2; material no. 03039773190. |
| Triglyceride reagent | Roche TRIGL Triglycerides; material no. 20767107322. |
| HDL-cholesterol reagent | Roche HDLC4 HDL-Cholesterol Gen.4; material no. 07528566190. |
| LDL-cholesterol reagent | Roche LDL-Cholesterol plus 2nd generation, configured for the cobas c 311. |
| Homocysteine reagent | Roche HCYS Homocysteine Enzymatic Assay (enzymatic cycling); material no. 05385415190. |
| Endothelin-1 ELISA | R&D Systems/Bio-Techne Endothelin-1 Quantikine ELISA Kit, catalogue no. DET100; 96-well sandwich ELISA for serum or plasma, read at 450 nm. |
| Nitric-oxide metabolite assay | Cayman Chemical Nitrate/Nitrite Colorimetric Assay Kit, item no. 780001; nitrate-reductase/Griess method, read at 540-550 nm. |
| Microplate reader | Thermo Scientific Multiskan FC, catalogue no. 51119000, fitted with 405-, 450- and 620-nm filters. |
| Calibration and quality control | Manufacturer-assigned calibrators, with at least two levels of internal quality-control material analysed daily in accordance with the laboratory standard operating procedure. |

Identifiers are transcribed from the laboratory equipment register, reagent inventory and kit packaging records held by the clinical laboratory. All chemistry analytes were measured on a single analyser platform; microplate assays were read on a single reader.

**Supplementary Table S11. Adverse-event definitions, severity grading and laboratory-safety criteria.**

| **Item** | **Definition applied** |
| --- | --- |
| Non-serious treatment-emergent adverse event | Any untoward medical occurrence arising after enrolment that did not meet a seriousness criterion; collected from enrolment through month 3. |
| Serious adverse event (ICH E2A) | Death; a life-threatening event; initial or prolonged hospitalization; persistent or substantial disability; congenital anomaly; or another medically important event requiring intervention. Collected through day 540. |
| Severity grade 1 | Mild; no intervention required. |
| Severity grade 2 | Moderate; minimal treatment required. |
| Severity grade 3 | Severe or medically significant. |
| Severity grade 4 | Life-threatening. |
| Severity grade 5 | Death. |
| Liver abnormality | Alanine aminotransferase or aspartate aminotransferase greater than 3 times the laboratory upper limit of normal. |
| Clinically important hyperbilirubinaemia | Total bilirubin greater than 2 times the laboratory upper limit of normal. |
| Renal abnormality | Serum creatinine greater than 1.5 times the participant’s baseline value. |
| Meaningful renal decline | Reduction in estimated glomerular filtration rate greater than 30% from baseline. |
| Supplement discontinuation because of an adverse event | Any event leading the investigator or the participant to stop spirulina permanently. |
| Treatment-related serious adverse event | A serious event adjudicated as possibly, probably or definitely related to the assigned strategy. |

Definitions are transcribed from the study safety manual. Seriousness follows ICH E2A. Severity grading and laboratory-safety criteria were applied by the treating clinical team and reviewed by the study investigators; causal attribution was adjudicated by the study investigators.

**Supplementary Table S12. Product-batch accountability, batch allocation by group and batch sensitivity analyses.**

| **Batch** | **Lot number** | **Expiry date** | **Dispensing period** | **Latest possible date of product use** | **Participants supplied** | **Participants receiving product after expiry** |
| --- | --- | --- | --- | --- | --- | --- |
| A | NIS21090450-SRL-09 | 31 Dec 2022 | Jun 2020 to Sep 2022 | Dec 2022 | 108 | 0 |
| B | SIM-SP-B12-20220915-02 | 30 Jun 2024 | Oct 2022 to Feb 2023 | May 2023 | 60 | 0 |
| C | SIM-SP-B12-20230220-01 | 31 Dec 2024 | Mar 2023 to Jun 2023 | Sep 2023 | 8 | 0 |
| Total |  |  | Jun 2020 to Jun 2023 | Sep 2023 | 176 | 0 |

Batch allocation by intervention group

| **Batch and dispensing period** | **Spirulina** | **Diet + spirulina** | **Total** |
| --- | --- | --- | --- |
| Batch A, Jun 2020 to Sep 2022 | 57 | 51 | 108 |
| Batch B, Oct to Nov 2022 | 24 | 21 | 45 |
| Batch B, Dec 2022 to Jan 2023 | 7 | 5 | 12 |
| Batch B, Feb 2023 | 0 | 3 | 3 |
| Batch C, Mar to Jun 2023 | 0 | 8 | 8 |
| Total | 88 | 88 | 176 |

Adherence, tolerability and 3-month outcomes by product batch (supplemented participants only)

| **Measure** | **Batch A (n=108)** | | **Batch B (n=60)** | | **Batch C (n=8)** |
| --- | --- | --- | --- | --- | --- |
| Mean 3-month spirulina adherence, % | 88.4 ± 8.5 | | 88.4 ± 8.4 | | 89.5 ± 6.5 |
| Adherence at least 80%, n (%) | 98 (90.7) | | 55 (91.7) | | 8 (100.0) |
| Any non-serious treatment-emergent adverse event, n (%) | 18 (16.7) | | 9 (15.0) | | 1 (12.5) |
| Gastrointestinal discomfort, n (%) | 7 (6.5) | | 3 (5.0) | | 1 (12.5) |
| **Outcome** | | **Adjusted difference for batches B and C versus batch A (95% CI)** | | **p value** | |
| Albumin, g/L | | +0.29 (-0.21 to +0.79) | | 0.253 | |
| Total protein, g/L | | -0.10 (-0.82 to +0.61) | | 0.779 | |
| Mini Nutritional Assessment, points | | -0.09 (-0.48 to +0.30) | | 0.658 | |
| Total cholesterol, mmol/L | | -0.02 (-0.11 to +0.08) | | 0.756 | |
| Triglycerides, mmol/L | | -0.01 (-0.07 to +0.05) | | 0.683 | |
| HDL cholesterol, mmol/L | | +0.03 (+0.00 to +0.05) | | 0.033 | |
| LDL cholesterol, mmol/L | | -0.05 (-0.15 to +0.05) | | 0.292 | |
| Homocysteine, µmol/L | | +0.04 (-0.20 to +0.27) | | 0.748 | |
| Nitric-oxide metabolites, µmol/L | | -1.36 (-2.29 to -0.44) | | 0.005 | |
| Endothelin-1, ng/L | | +0.12 (-1.80 to +2.04) | | 0.900 | |

Lot numbers, expiry dates and dispensing periods are transcribed from the hospital pharmacy dispensing log and the signed certificates of analysis held on file. Batch A is the lot described in the previous version of this report; Batches B and C were procured as the first batch was exhausted and were not previously described. Because each participant received a single 3-month supply at the point of dispensing, the latest possible date of product use is the dispensing end date plus 3 months; for every batch this date precedes the labelled expiry date, so no participant received or consumed product beyond its shelf life. Batch effects on 3-month outcomes were estimated among supplemented participants only, adjusting for the baseline value of the outcome, group and the full measured-confounder set, with heteroscedasticity-consistent (HC3) standard errors. Two of the ten contrasts reached nominal significance in opposite directions and neither survived Benjamini-Hochberg correction at the 5% level; the Fisher combined p value across the ten outcomes was 0.156. The nominal nitric-oxide difference favoured the oldest batch, which is the opposite of what product degradation would produce.

**Supplementary Table S13. Enrolment timeline by group and definition of the concurrent-recruitment cohort.**

| **Enrolment period** | **Control** | | **Spirulina** | | **Diet + spirulina** | | **Total** | | **All three groups open** |
| --- | --- | --- | --- | --- | --- | --- | --- | --- | --- |
| Jun 2020 to Sep 2022 | 68 | | 57 | | 51 | | 176 | | Yes |
| Oct to Nov 2022 | 20 | | 24 | | 21 | | 65 | | Yes |
| Dec 2022 to Jan 2023 | 0 | | 7 | | 5 | | 12 | | No |
| Feb 2023 | 0 | | 0 | | 3 | | 3 | | No |
| Mar to Jun 2023 | 0 | | 0 | | 8 | | 8 | | No |
| Total | 88 | | 88 | | 88 | | 264 | |  |
| **Analysis population** | | **Control** | | **Spirulina** | | **Diet + spirulina** | | **Total** | |
| Full cohort | | 88 | | 88 | | 88 | | 264 | |
| Enrolled during the concurrent recruitment window | | 88 | | 81 | | 72 | | 241 | |
| Excluded because enrolled after Nov 2022 | | 0 | | 7 | | 16 | | 23 | |
| Complete paired 3-month biomarker data, concurrent window | | 86 | | 80 | | 70 | | 236 | |
| Included in the concurrent time-to-event analysis | | 88 | | 81 | | 72 | | 241 | |

Quota closure dates were 22 November 2022 for control, 31 January 2023 for spirulina and 9 June 2023 for diet plus spirulina. The concurrent recruitment window is defined as the interval during which all three group quotas were simultaneously open, namely June 2020 to November 2022. Twenty-three intervention participants were enrolled after the control quota closed and therefore had no contemporaneously recruited control comparators. The timeline is displayed in Supplementary Figure S6.

**Supplementary Table S14. Sensitivity analysis restricted to the concurrent recruitment window.**

| **Outcome** | **Spirulina vs control, full cohort (95% CI)** | | **Spirulina vs control, concurrent window (95% CI)** | | **Diet + spirulina vs control, full cohort (95% CI)** | | **Diet + spirulina vs control, concurrent window (95% CI)** |
| --- | --- | --- | --- | --- | --- | --- | --- |
| Albumin, g/L | +0.83 (+0.45 to +1.21) | | +0.86 (+0.48 to +1.24) | | +1.09 (+0.67 to +1.51) | | +1.12 (+0.66 to +1.57) |
| Total protein, g/L | +1.23 (+0.68 to +1.77) | | +1.27 (+0.71 to +1.84) | | +1.92 (+1.29 to +2.55) | | +1.81 (+1.13 to +2.49) |
| Mini Nutritional Assessment, points | +0.89 (+0.59 to +1.19) | | +0.89 (+0.59 to +1.20) | | +1.60 (+1.26 to +1.95) | | +1.73 (+1.39 to +2.08) |
| Total cholesterol, mmol/L | -0.10 (-0.18 to -0.03) | | -0.10 (-0.18 to -0.02) | | -0.26 (-0.34 to -0.17) | | -0.26 (-0.35 to -0.18) |
| Triglycerides, mmol/L | -0.09 (-0.14 to -0.03) | | -0.09 (-0.14 to -0.03) | | -0.20 (-0.25 to -0.15) | | -0.18 (-0.24 to -0.13) |
| HDL cholesterol, mmol/L | +0.05 (+0.03 to +0.07) | | +0.05 (+0.03 to +0.07) | | +0.06 (+0.04 to +0.09) | | +0.06 (+0.04 to +0.09) |
| LDL cholesterol, mmol/L | -0.12 (-0.20 to -0.04) | | -0.12 (-0.20 to -0.04) | | -0.25 (-0.33 to -0.17) | | -0.26 (-0.34 to -0.18) |
| Homocysteine, µmol/L | -0.52 (-0.74 to -0.31) | | -0.49 (-0.71 to -0.28) | | -1.05 (-1.29 to -0.81) | | -1.07 (-1.31 to -0.82) |
| Nitric-oxide metabolites, µmol/L | +1.99 (+1.17 to +2.82) | | +2.07 (+1.21 to +2.93) | | +3.65 (+2.73 to +4.56) | | +3.71 (+2.72 to +4.70) |
| Endothelin-1, ng/L | -2.18 (-3.78 to -0.58) | | -1.98 (-3.62 to -0.33) | | -5.66 (-7.48 to -3.84) | | -5.67 (-7.63 to -3.71) |
| **Time-to-event measure** | | **Control** | | **Spirulina** | | **Diet + spirulina** | |
| Participants, full cohort | | 88 | | 88 | | 88 | |
| First MACE, full cohort | | 24 | | 18 | | 16 | |
| Adjusted hazard ratio, full cohort | | Reference | | 0.74 (0.40 to 1.37) | | 0.62 (0.33 to 1.17) | |
| Participants, concurrent window | | 88 | | 81 | | 72 | |
| First MACE, concurrent window | | 24 | | 17 | | 13 | |
| First MACE among excluded later recruits | | 0 | | 1 | | 3 | |
| Adjusted hazard ratio, concurrent window | | Reference | | 0.75 (0.40 to 1.40) | | 0.63 (0.32 to 1.24) | |

Biomarker models are identical in specification to the primary analysis, namely ordinary least squares regression on the baseline value of the outcome and the full measured clinical and treatment-selection covariate set with HC3 standard errors, refitted after restriction to participants enrolled during the concurrent recruitment window. All 20 restricted contrasts with control retained significance after Benjamini-Hochberg correction. The Cox model adjusted for age, sex, prior myocardial infarction, left-ventricular ejection fraction and baseline LDL cholesterol. Estimates are shown graphically in Supplementary Figure S7.

**Supplementary Table S15. Reconciliation of serious adverse events: participants and episodes.**

| **Event-accounting measure** | **Control** | **Spirulina** | **Diet + spirulina** |
| --- | --- | --- | --- |
| Participants with a first MACE | 24 | 18 | 16 |
| First MACE episodes | 24 | 18 | 16 |
| Recurrent MACE episodes | 2 | 2 | 2 |
| Total MACE episodes | 26 | 20 | 18 |
| Participants with a non-MACE serious adverse event | 2 | 1 | 2 |
| Non-MACE serious-event episodes | 2 | 1 | 2 |
| Total serious-event episodes | 28 | 21 | 20 |
| Participants with both a MACE and a non-MACE serious event | 1 | 0 | 1 |
| Unique participants with any serious event | 25 | 19 | 17 |
| Unique participants with any serious event, % | 28.4 | 21.6 | 19.3 |
| Observed person-years | 104.3 | 109.8 | 111.3 |
| Serious-event episodes per 100 person-years | 26.9 | 19.1 | 18.0 |
| Risk difference versus control for participants with any serious event, percentage points (95% CI) | Reference | -6.8 (-19.3 to +6.0) | -9.1 (-21.4 to +3.5) |

The arithmetic reconciles as follows. Control: 24 first MACE episodes plus 2 recurrent MACE episodes plus 2 non-MACE serious-event episodes equals 28 episodes. Spirulina: 18 plus 2 plus 1 equals 21 episodes. Diet plus spirulina: 16 plus 2 plus 2 equals 20 episodes. The number of unique participants is smaller than the number of episodes because 2 participants per group contributed a recurrent MACE episode, and because one control participant and one combined-group participant experienced both a MACE and a non-MACE serious event. Risk differences use the Newcombe hybrid-score method without multiplicity adjustment and are descriptive. Only first MACE contributes to the time-to-event analyses reported in Table 3, in keeping with the prespecified first-event endpoint definition. The accounting is shown graphically in Supplementary Figure S8.

**Supplementary figure legends for the figures added at this revision**

Supplementary Figure S6. Enrolment timeline and product-batch accountability. Panel A shows the number of participants enrolled in each group within each enrolment period; the shaded region marks the concurrent recruitment window during which all three group quotas were open. Panel B shows, for each product batch, the dispensing period, the residual period of 3-month product use after the last dispensing, and the labelled expiry date. For every batch the residual use period ends before the expiry marker.

Supplementary Figure S7. Adjusted 3-month between-group differences in the full cohort and in the concurrent-recruitment cohort. Open circles denote full-cohort estimates and filled squares denote concurrent-window estimates, with horizontal bars showing 95% confidence intervals. The horizontal axis uses a symmetric logarithmic scale because the ten outcomes are expressed in different units.

Supplementary Figure S8. Accounting of serious adverse events. Panel A decomposes the total serious-event episode count in each group into first MACE episodes, recurrent MACE episodes and non-MACE serious-event episodes. Panel B contrasts the number of unique participants experiencing at least one serious event with the total number of serious-event episodes.
