# Supplementary material for: Three-month biomarker changes after spirulina with or without nursing-led dietary management in older adults with coronary artery disease: a prospective nonrandomized comparative study
Source: Front Med (Lausanne). 2026 Aug 14;13:1910826. doi: 10.3389/fmed.2026.1910826 (PMC13522770; doi:10.3389/fmed.2026.1910826)
Supplement: Supplementary file 2 [file Data_Sheet_2.docx]

from __future__ import annotations

import json, math, warnings

from pathlib import Path

import numpy as np

import pandas as pd

from scipy import stats

import statsmodels.api as sm

from statsmodels.duration.hazard_regression import PHReg

from sklearn.preprocessing import StandardScaler

from sklearn.linear_model import LogisticRegression

from sklearn.pipeline import make_pipeline

import matplotlib.pyplot as plt

from PIL import Image

from matplotlib.patches import FancyBboxPatch, FancyArrowPatch

warnings.filterwarnings('ignore')

SCRIPT_DIR = Path(__file__).resolve().parent

DATA_FILENAME = 'Supplementary Data File 1 Deidentified Dataset.csv'

INPUT_CSV = SCRIPT_DIR / DATA_FILENAME

if not INPUT_CSV.is_file():

raise FileNotFoundError(

f"Required dataset not found: {INPUT_CSV}\n"

f"Place '{DATA_FILENAME}' in the same folder as this analysis script."

)

OUT = SCRIPT_DIR / 'Reproduced_Outputs'

FIG=OUT/'Figures'

SUPFIG=OUT/'Supplementary_Figures'

AN=OUT/'Analysis'

for p in [OUT,FIG,SUPFIG,AN]: p.mkdir(parents=True,exist_ok=True)

df=pd.read_csv(INPUT_CSV)

cat_cols={'id','group','group_label','statin_type','biomarker_missing_reason','baseline_lab_batch','month3_lab_batch','MACE_type'}

for _c in df.columns:

if _c not in cat_cols:

df[_c]=pd.to_numeric(df[_c],errors='coerce')

G=['CG','SP','DS']

LABEL={'CG':'Conventional nursing','SP':'Spirulina','DS':'Diet control + spirulina'}

SHORT={'CG':'Control','SP':'Spirulina','DS':'Diet + spirulina'}

\# The distributed dataset intentionally excludes exact enrolment dates.

\# Only the broad, non-identifying `calendar_period` category is retained.

\# outcome metadata

outs={

'ALB':('ALB_baseline_g_L','ALB_3m_g_L','Albumin','g/L',2),

'TP':('TP_baseline_g_L','TP_3m_g_L','Total protein','g/L',2),

'MNA':('MNA_baseline_0_30','MNA_3m_0_30','Mini Nutritional Assessment','points',2),

'TC':('TC_baseline_mmol_L','TC_3m_mmol_L','Total cholesterol','mmol/L',2),

'TG':('TG_baseline_mmol_L','TG_3m_mmol_L','Triglycerides','mmol/L',2),

'HDL':('HDL_baseline_mmol_L','HDL_3m_mmol_L','HDL cholesterol','mmol/L',2),

'LDL':('LDL_baseline_mmol_L','LDL_3m_mmol_L','LDL cholesterol','mmol/L',2),

'Hcy':('Hcy_baseline_umol_L','Hcy_3m_umol_L','Homocysteine','µmol/L',2),

'NO':('NO_baseline_umol_L','NO_3m_umol_L','Nitric oxide metabolites','µmol/L',2),

'ET1':('ET1_baseline_ng_L','ET1_3m_ng_L','Endothelin-1','ng/L',2),

}

full_cov=['age_years','male','BMI_kg_m2','current_smoker','hypertension','diabetes','prior_MI','prior_PCI_CABG','LVEF_pct','multivessel_CAD','CCS_angina_class','family_support_adequate','supplement_willingness','programme_capacity_available','education_high_school_plus','low_SES','health_literacy_score_1_9','baseline_diet_quality_score_1_8','calendar_period','high_intensity_statin_baseline','medication_adherence_pct']

minimal_cov=['age_years','male','BMI_kg_m2']

extended_cov=full_cov+['index_contact_within_7d','ACS_at_index','statin_new_or_intensified','high_intensity_statin_3m','eGFR_mL_min_1_73m2','hsCRP_mg_L','high_ward_workload']

def bh(p):

p=np.asarray(p,float); n=len(p); order=np.argsort(p); q=np.empty(n); prev=1

for j in range(n-1,-1,-1):

i=order[j]; prev=min(prev,p[i]*n/(j+1)); q[i]=prev

return q

def fit_ancova(outcome_key,covs,subset=None,add_batch=False,weights=None):

b,m,_,_,_=outs[outcome_key]

d=df[df.biomarker_complete.eq(1)].copy()

if subset is not None: d=d.loc[subset(d)].copy()

cols=[b]+covs

if add_batch:

\# Batch/plate indicators are sensitivity terms; plate only for ELISA-like outcomes

cols += ['month3_lab_batch']

if outcome_key in ['NO','ET1']: cols += ['ELISA_plate_number']

X=pd.DataFrame({'const':1.0,'SP':d.group.eq('SP').astype(int),'DS':d.group.eq('DS').astype(int)},index=d.index)

for c in cols:

if c in ['month3_lab_batch']:

z=pd.get_dummies(d[c].astype(str),prefix=c,drop_first=True,dtype=float)

X=pd.concat([X,z],axis=1)

else:

X[c]=pd.to_numeric(d[c],errors='coerce')

y=d[m].astype(float)

mask=X.notna().all(axis=1)&y.notna()

X=X.loc[mask]; y=y.loc[mask]

if weights is None:

fit=sm.OLS(y,X).fit(cov_type='HC3')

else:

w=pd.Series(weights,index=df.index).loc[X.index]

fit=sm.WLS(y,X,weights=w).fit(cov_type='HC3')

def contrast(vec):

tt=fit.t_test(vec)

est=float(np.asarray(tt.effect).squeeze()); se=float(np.asarray(tt.sd).squeeze())

return {'estimate':est,'ci_low':est-1.96*se,'ci_high':est+1.96*se,'p':float(tt.pvalue)}

v1=np.zeros(X.shape[1]); v1[X.columns.get_loc('SP')]=1

v2=np.zeros(X.shape[1]); v2[X.columns.get_loc('DS')]=1

v3=v2-v1

return {'SP-CG':contrast(v1),'DS-CG':contrast(v2),'DS-SP':contrast(v3),'n':int(len(y)),'r2':float(fit.rsquared)}

\# Generalized overlap weights from regularized multinomial logit

balance_vars=['age_years','male','BMI_kg_m2','current_smoker','hypertension','diabetes','dyslipidaemia','prior_MI','prior_PCI_CABG','CAD_duration_years','LVEF_pct','multivessel_CAD','CCS_angina_class','index_contact_within_7d','ACS_at_index','family_support_adequate','supplement_willingness','programme_capacity_available','education_high_school_plus','low_SES','health_literacy_score_1_9','baseline_diet_quality_score_1_8','high_ward_workload','high_intensity_statin_baseline','medication_adherence_pct','antiplatelet','ACEI_ARB','beta_blocker','eGFR_mL_min_1_73m2','hsCRP_mg_L','LDL_baseline_mmol_L','Hcy_baseline_umol_L']

Xps=df[balance_vars+['calendar_period']].astype(float).values

yps=np.array([G.index(x) for x in df.group])

ps_model=make_pipeline(StandardScaler(),LogisticRegression(C=0.1,max_iter=10000,solver='lbfgs'))

ps_model.fit(Xps,yps)

ps=np.clip(ps_model.predict_proba(Xps),1e-6,1-1e-6); ps=ps/ps.sum(1,keepdims=True)

h=1/np.sum(1/ps,axis=1)

ow=np.array([h[i]/ps[i,yps[i]] for i in range(len(df))])

\# Normalize within group to mean 1 for interpretable diagnostics

for k,g in enumerate(G): ow[yps==k] /= ow[yps==k].mean()

df['revised_overlap_weight']=ow

def wmean(x,w): return float(np.sum(x*w)/np.sum(w))

def wvar(x,w):

m=wmean(x,w); return float(np.sum(w*(x-m)**2)/np.sum(w))

def max_pair_smd(var,w=None):

arr=[]

for i,a in enumerate(G):

for b in G[i+1:]:

da=df[df.group.eq(a)]; db=df[df.group.eq(b)]

xa=da[var].astype(float).values; xb=db[var].astype(float).values

if w is None:

ma,mb=xa.mean(),xb.mean(); va,vb=xa.var(ddof=1),xb.var(ddof=1)

else:

wa=w[da.index]; wb=w[db.index]

ma,mb=wmean(xa,wa),wmean(xb,wb); va,vb=wvar(xa,wa),wvar(xb,wb)

den=math.sqrt((va+vb)/2)

arr.append(abs(ma-mb)/den if den else 0)

return max(arr)

def group_stat(var,g,w=None):

d=df[df.group.eq(g)]; x=d[var].astype(float).values

if w is None: return float(x.mean())

return wmean(x,w[d.index])

balance=[]

for v in balance_vars+['calendar_period']:

balance.append({'variable':v,**{f'{g}_unweighted':group_stat(v,g) for g in G},**{f'{g}_weighted':group_stat(v,g,ow) for g in G},'max_smd_before':max_pair_smd(v),'max_smd_after':max_pair_smd(v,ow),'missing_n':int(df[v].isna().sum())})

balance_df=pd.DataFrame(balance)

weight_diag=[]

for g in G:

w=ow[df.group.eq(g)]

weight_diag.append({'group':g,'n':int(len(w)),'min':float(w.min()),'p1':float(np.quantile(w,.01)),'median':float(np.median(w)),'mean':float(w.mean()),'p99':float(np.quantile(w,.99)),'max':float(w.max()),'ess':float(w.sum()**2/np.sum(w*w))})

weight_diag_df=pd.DataFrame(weight_diag)

\# Main outcomes

summ=[]; anc={}; p20=[]; keys=[]

for k,(b,m,name,unit,dec) in outs.items():

row={'outcome':k,'label':name,'unit':unit}

for g in G:

d=df[df.group.eq(g)]

row[f'{g}_baseline_n']=int(d[b].notna().sum()); row[f'{g}_baseline_mean']=float(d[b].mean()); row[f'{g}_baseline_sd']=float(d[b].std(ddof=1))

row[f'{g}_3m_n']=int(d[m].notna().sum()); row[f'{g}_3m_mean']=float(d[m].mean()); row[f'{g}_3m_sd']=float(d[m].std(ddof=1))

anc[k]=fit_ancova(k,full_cov)

for c in ['SP-CG','DS-CG']:

p20.append(anc[k][c]['p']); keys.append((k,c))

summ.append(row)

q20=bh(p20)

for (k,c),q in zip(keys,q20): anc[k][c]['q']=float(q)

outcome_df=pd.DataFrame(summ)

\# Sensitivities

sensitivity=[]

for k in outs:

specs={

'Minimal adjustment': fit_ancova(k,minimal_cov),

'Full measured-confounder adjustment': anc[k],

'Extended clinical adjustment': fit_ancova(k,extended_cov),

'Exclude statin initiation/intensification': fit_ancova(k,full_cov,subset=lambda d:d.statin_new_or_intensified.eq(0)),

'Exclude acute coronary syndrome at index': fit_ancova(k,full_cov,subset=lambda d:d.ACS_at_index.eq(0)),

'Laboratory batch/plate adjusted': fit_ancova(k,full_cov,add_batch=True),

'Generalized-overlap weighted': fit_ancova(k,[outs[k][0]],weights=ow),

}

for spec,res in specs.items():

for c in ['SP-CG','DS-CG','DS-SP']:

sensitivity.append({'outcome':k,'analysis':spec,'contrast':c,**res[c],'n':res['n']})

sens_df=pd.DataFrame(sensitivity)

\# KM and Aalen-Johansen

def km_curve(group):

d=df[df.group.eq(group)].sort_values('event_or_censor_day')

times=np.sort(d.event_or_censor_day.unique())

n=len(d); S=1.; out=[(0,S,n,0,0)]

for t in times:

at=int((d.event_or_censor_day>=t).sum()); ev=int(((d.event_or_censor_day==t)&(d.MACE_event==1)).sum()); cen=int(((d.event_or_censor_day==t)&(d.MACE_event==0)).sum())

if ev: S*=1-ev/at

out.append((float(t),float(S),at-ev-cen,ev,cen))

return out

def step_at(curve,t):

s=curve[0][1]

for row in curve:

if row[0]<=t:s=row[1]

else:break

return s

def aj_curve(group):

d=df[df.group.eq(group)].sort_values('event_or_censor_day')

times=np.sort(d.event_or_censor_day.unique())

S=1.; F1=0.; F2=0.; out=[(0,S,F1,F2)]

for t in times:

at=int((d.event_or_censor_day>=t).sum())

d1=int(((d.event_or_censor_day==t)&(d.MACE_event==1)).sum())

d2=int(((d.event_or_censor_day==t)&(d.noncardiac_death==1)).sum())

if at>0:

F1+=S*d1/at; F2+=S*d2/at; S*=1-(d1+d2)/at

out.append((float(t),float(S),float(F1),float(F2)))

return out

km={g:km_curve(g) for g in G}; aj={g:aj_curve(g) for g in G}

def bootstrap_km_rd(B=500,seed=20260723):

rng=np.random.default_rng(seed); vals={('SP','CG'):[],('DS','CG'):[],('DS','SP'):[]}

groups={g:df[df.group.eq(g)] for g in G}

for _ in range(B):

risks={}

for g,d in groups.items():

idx=rng.integers(0,len(d),len(d)); dd=d.iloc[idx].copy()

\# local KM

S=1.

for t in np.sort(dd.event_or_censor_day.unique()):

at=int((dd.event_or_censor_day>=t).sum()); ev=int(((dd.event_or_censor_day==t)&(dd.MACE_event==1)).sum())

if ev and at:S*=1-ev/at

risks[g]=1-S

vals[('SP','CG')].append(risks['SP']-risks['CG']); vals[('DS','CG')].append(risks['DS']-risks['CG']); vals[('DS','SP')].append(risks['DS']-risks['SP'])

return {f'{a}-{b}':{'estimate':(1-step_at(km[a],540))-(1-step_at(km[b],540)),'ci_low':float(np.quantile(x,.025)),'ci_high':float(np.quantile(x,.975))} for (a,b),x in vals.items()}

rd=bootstrap_km_rd()

\# Cox model and stability

cox_cov=['age_years','male','prior_MI','LVEF_pct','LDL_baseline_mmol_L']

def cox_fit(data,covs=cox_cov):

X=pd.DataFrame({'SP':data.group.eq('SP').astype(int),'DS':data.group.eq('DS').astype(int)},index=data.index)

for c in covs: X[c]=data[c].astype(float)

fit=PHReg(data.event_or_censor_day.astype(float),X,status=data.MACE_event.astype(int),ties='efron').fit(disp=0)

out={}

for i,n in enumerate(X.columns):

out[n]={'hr':float(np.exp(fit.params[i])),'ci_low':float(np.exp(fit.params[i]-1.96*fit.bse[i])),'ci_high':float(np.exp(fit.params[i]+1.96*fit.bse[i])),'p':float(fit.pvalues[i])}

return fit,X,out

cox_fit_obj,cox_X,cox=cox_fit(df)

\# PH diagnostic: correlation of Schoenfeld residual with log event time

sr=cox_fit_obj.schoenfeld_residuals; eventmask=df.MACE_event.eq(1).values; et=np.log(df.loc[eventmask,'event_or_censor_day'].astype(float).values)

ph=[]

for j,n in enumerate(cox_X.columns):

r,p=stats.pearsonr(et,sr[eventmask,j]); ph.append({'term':n,'correlation':float(r),'p':float(p)})

ph_df=pd.DataFrame(ph); global_ph=float(stats.combine_pvalues(ph_df.p.values,method='fisher').pvalue)

\# bootstrap HR distribution stratified by group

rng=np.random.default_rng(20260723); boot=[]

for b in range(100):

dd=pd.concat([df[df.group.eq(g)].iloc[rng.integers(0,88,88)] for g in G],ignore_index=True)

try:

_,_,o=cox_fit(dd)

boot.append({'SP':o['SP']['hr'],'DS':o['DS']['hr']})

except Exception: pass

boot_df=pd.DataFrame(boot)

cox_boot={g:{'median':float(boot_df[g].median()),'ci_low':float(boot_df[g].quantile(.025)),'ci_high':float(boot_df[g].quantile(.975)),'successful_resamples':int(len(boot_df))} for g in ['SP','DS']}

\# MACE table

mace=[]

for g in G:

d=df[df.group.eq(g)]; kmi=1-step_at(km[g],540); aji=aj[g][-1][2]

mace.append({'group':g,'n':88,'MACE_n':int(d.MACE_event.sum()),'MI_n':int((d.MACE_type=='MI').sum()),'stroke_n':int((d.MACE_type=='Ischaemic stroke').sum()),'cardiac_death_n':int((d.MACE_type=='Cardiac death').sum()),'noncardiac_death_n':int(d.noncardiac_death.sum()),'lost_n':int(d.lost_to_followup.sum()),'KM_risk_18m':float(kmi),'AJ_CIF_18m':float(aji),'cox_hr':1.0 if g=='CG' else cox[g]['hr'],'cox_ci_low':1.0 if g=='CG' else cox[g]['ci_low'],'cox_ci_high':1.0 if g=='CG' else cox[g]['ci_high'],'cox_p':np.nan if g=='CG' else cox[g]['p']})

mace_df=pd.DataFrame(mace)

\# Safety and adherence

safety_vars=[('GI_discomfort','Gastrointestinal discomfort'),('rash_allergy','Rash/allergy'),('abnormal_LFT','Abnormal liver-function test'),('renal_AE','Renal adverse event'),('other_nonserious_TEAE','Other non-serious adverse event'),('any_nonserious_TEAE','Any non-serious treatment-emergent adverse event'),('supplement_discontinued','Supplement discontinued because of adverse event'),('nonMACE_SAE','Non-MACE serious adverse event'),('all_SAE_including_MACE','All serious adverse events, including MACE'),('treatment_related_SAE','Treatment-related serious adverse event')]

safety=[]

for v,l in safety_vars:

row={'outcome':l}

for g in G:

n=int(df.loc[df.group.eq(g),v].sum()); row[f'{g}_n']=n; row[f'{g}_pct']=100*n/88

safety.append(row)

safety_df=pd.DataFrame(safety)

adherence=[]

for g in G:

d=df[df.group.eq(g)]

sa=pd.to_numeric(d.spirulina_adherence_pct,errors='coerce'); sg=pd.to_numeric(d.spirulina_adherence_ge80,errors='coerce'); dc=pd.to_numeric(d.diet_checklist_completion_pct,errors='coerce'); c6=pd.to_numeric(d.continued_spirulina_m6,errors='coerce'); c12=pd.to_numeric(d.continued_spirulina_m12,errors='coerce'); c18=pd.to_numeric(d.continued_spirulina_m18,errors='coerce')

adherence.append({'group':g,'spirulina_adherence_mean':float(sa.mean()) if sa.notna().any() else np.nan,'spirulina_ge80_n':int(sg.fillna(0).sum()),'diet_checklist_mean':float(dc.mean()) if dc.notna().any() else np.nan,'diet_checklist_ge80_n':int((dc>=80).sum()),'continued_m6_n':int(c6.fillna(0).sum()),'continued_m12_n':int(c12.fillna(0).sum()),'continued_m18_n':int(c18.fillna(0).sum())})

adherence_df=pd.DataFrame(adherence)

\# Clinical-setting table

setting=[]

for g in G:

d=df[df.group.eq(g)]

setting.append({'group':g,'index_contact_within_7d_n':int(d.index_contact_within_7d.sum()),'ACS_at_index_n':int(d.ACS_at_index.sum()),'days_index_to_baseline_median':float(d.days_index_to_baseline.median()),'days_index_to_baseline_q1':float(d.days_index_to_baseline.quantile(.25)),'days_index_to_baseline_q3':float(d.days_index_to_baseline.quantile(.75)),'statin_new_or_intensified_n':int(d.statin_new_or_intensified.sum()),'high_intensity_statin_baseline_n':int(d.high_intensity_statin_baseline.sum()),'high_intensity_statin_3m_n':int(d.high_intensity_statin_3m.sum()),'calendar_period_min':int(d.calendar_period.min()),'calendar_period_max':int(d.calendar_period.max())})

setting_df=pd.DataFrame(setting)

\# Diet process table, DS only

dd=df[df.group.eq('DS')]

diet=[]

for v,l in [('diet_salt_process_met','Salt target documented'),('diet_cholesterol_process_met','Cholesterol target documented'),('diet_fat_process_met','Fat target documented'),('diet_protein_process_met','Protein target documented'),('diet_carbohydrate_process_met','Carbohydrate target documented'),('diet_fibre_process_met','Fibre/produce target documented')]:

diet.append({'process':l,'n':int(dd[v].sum()),'pct':100*float(dd[v].mean())})

diet_df=pd.DataFrame(diet)

\# Missingness

missing=[]

for g in G:

d=df[df.group.eq(g)]

missing.append({'group':g,'complete_n':int(d.biomarker_complete.sum()),'missing_n':int((1-d.biomarker_complete).sum()),'withdrew_consent_n':int((d.biomarker_missing_reason=='Withdrew consent').sum()),'relocated_n':int((d.biomarker_missing_reason=='Relocated').sum())})

missing_df=pd.DataFrame(missing)

\# Table 1 main selected variables

main_vars=[

('age_years','Age, years','cont'),('male','Male sex','bin'),('BMI_kg_m2','Body mass index, kg/m²','cont'),('current_smoker','Current smoker','bin'),('hypertension','Hypertension','bin'),('diabetes','Diabetes','bin'),('prior_MI','Previous myocardial infarction','bin'),('prior_PCI_CABG','Previous PCI/CABG','bin'),('LVEF_pct','Left ventricular ejection fraction, %','cont'),('multivessel_CAD','Multivessel coronary disease','bin'),('CCS_angina_class','CCS angina class','cont'),('ACS_at_index','Acute coronary syndrome at index contact','bin'),('family_support_adequate','Adequate family support','bin'),('supplement_willingness','Willingness to take supplementation','bin'),('programme_capacity_available','Dietary-programme capacity available','bin'),('education_high_school_plus','High-school education or higher','bin'),('low_SES','Low socioeconomic status','bin'),('health_literacy_score_1_9','Health-literacy score (1–9)','cont'),('baseline_diet_quality_score_1_8','Baseline diet-quality score (1–8)','cont'),('medication_adherence_pct','Medication adherence, %','cont'),('high_intensity_statin_baseline','High-intensity statin at baseline','bin'),('eGFR_mL_min_1_73m2','Estimated GFR, mL/min/1.73 m²','cont'),('hsCRP_mg_L','High-sensitivity C-reactive protein, mg/L','cont')]

t1=[]

for v,l,t in main_vars:

row={'variable':v,'label':l,'type':t}

for g in G:

d=df[df.group.eq(g)]

if t=='bin': row[g]=f"{int(d[v].sum())} ({100*d[v].mean():.1f})"

else: row[g]=f"{d[v].mean():.2f} ± {d[v].std(ddof=1):.2f}"

row['max_smd_before']=max_pair_smd(v); row['max_smd_after']=max_pair_smd(v,ow)

t1.append(row)

t1_df=pd.DataFrame(t1)

\# Export CSVs and analysis JSON

exports={'Table1_Baseline.csv':t1_df,'Table2_Biomarkers.csv':outcome_df,'Table3_MACE.csv':mace_df,'Table4_Safety.csv':safety_df,'TableS3a_Full_Balance.csv':balance_df,'TableS3b_Weight_Diagnostics.csv':weight_diag_df,'TableS4_Adherence.csv':adherence_df,'TableS5_Sensitivity.csv':sens_df,'TableS6_PH_Diagnostics.csv':ph_df,'TableS7_Diet_Process.csv':diet_df,'TableS8_Clinical_Setting.csv':setting_df,'TableS9_Missingness.csv':missing_df}

for fn,x in exports.items(): x.to_csv(AN/fn,index=False)

\# deidentified analysis dataset and dictionary

df.to_csv(AN/'Supplementary_Data_File_1_Deidentified_Dataset.csv',index=False)

dictionary=[]

for c in df.columns:

dictionary.append({'variable':c,'dtype':str(df[c].dtype),'missing_n':int(df[c].isna().sum()),'example_or_range': (f"{df[c].min()} to {df[c].max()}" if pd.api.types.is_numeric_dtype(df[c]) else ', '.join(map(str,df[c].dropna().astype(str).unique()[:5])))})

pd.DataFrame(dictionary).to_csv(AN/'Data_Dictionary.csv',index=False)

report={'counts':{g:{'n':88,'biomarker_complete':int(df.loc[df.group.eq(g),'biomarker_complete'].sum()),'MACE':int(df.loc[df.group.eq(g),'MACE_event'].sum()),'noncardiac_death':int(df.loc[df.group.eq(g),'noncardiac_death'].sum()),'lost':int(df.loc[df.group.eq(g),'lost_to_followup'].sum()),'complete_endpoint':int(df.loc[df.group.eq(g),'complete_18m_endpoint_ascertainment'].sum())} for g in G},'ancova':anc,'risk_differences':rd,'cox':cox,'cox_bootstrap':cox_boot,'ph_global_fisher_p':global_ph,'weights':weight_diag,'max_smd_before':float(balance_df.max_smd_before.max()),'max_smd_after':float(balance_df.max_smd_after.max()),'km_risk_18m':{g:float(1-step_at(km[g],540)) for g in G},'aj_cif_18m':{g:float(aj[g][-1][2]) for g in G},'risksets':{g:{str(t):int((df.loc[df.group.eq(g),'event_or_censor_day']>=t).sum()) for t in [0,90,180,270,360,450,540]} for g in G},'early_censor_intervals':{g:{f'{lo}-{hi}':int(((df.group.eq(g))&(df.MACE_event.eq(0))&(df.event_or_censor_day>lo)&(df.event_or_censor_day<=hi)&(df.event_or_censor_day<540)).sum()) for lo,hi in zip([0,90,180,270,360,450],[90,180,270,360,450,540])} for g in G}}

(AN/'Final_Analysis_Report.json').write_text(json.dumps(report,indent=2),encoding='utf-8')

\# Figures helpers

plt.rcParams.update({'font.size':8,'axes.labelsize':9,'legend.fontsize':7,'xtick.labelsize':7,'ytick.labelsize':7,'lines.linewidth':1.6,'figure.dpi':150})

def savefig(fig,name,folder=FIG):

\# Save an RGB, LZW-compressed 600-dpi TIFF for journal submission and a PNG preview.

tmp=folder/f'.{name}_rgba.tiff'

out_tif=folder/f'{name}.tiff'

fig.savefig(tmp,dpi=600,bbox_inches='tight',facecolor='white',transparent=False,

pil_kwargs={'compression':'tiff_lzw'})

with Image.open(tmp) as im:

im.convert('RGB').save(out_tif,dpi=(600,600),compression='tiff_lzw')

tmp.unlink(missing_ok=True)

fig.savefig(folder/f'{name}.png',dpi=220,bbox_inches='tight',facecolor='white',transparent=False)

plt.close(fig)

\# Figure 1 flow diagram

fig,ax=plt.subplots(figsize=(10.0,6.25)); ax.set_xlim(0,1); ax.set_ylim(0,1); ax.axis('off')

def box(x,y,w,h,text,fs=9.0,weight='normal'):

p=FancyBboxPatch((x,y),w,h,boxstyle='round,pad=0.008,rounding_size=0.006',

facecolor='white',edgecolor='black',linewidth=1.15)

ax.add_patch(p)

ax.text(x+w/2,y+h/2,text,ha='center',va='center',fontsize=fs,fontweight=weight,

linespacing=1.12)

return (x,y,w,h)

def arrow(a,b,start_x=None,end_x=None):

x1=a[0]+a[2]/2 if start_x is None else start_x

y1=a[1]

x2=b[0]+b[2]/2 if end_x is None else end_x

y2=b[1]+b[3]

ax.add_patch(FancyArrowPatch((x1,y1),(x2,y2),arrowstyle='-|>',mutation_scale=10,

linewidth=1.0,shrinkA=1.5,shrinkB=1.5))

top=box(.31,.89,.38,.07,'Formally screened while the relevant\ngroup quota remained open (n = 342)',fs=10.2)

ex=box(.33,.76,.34,.07,'Excluded (n = 78)\nIneligible, declined, or incomplete baseline data',fs=9.2)

inc=box(.34,.64,.32,.062,'Enrolled at time zero\n(n = 264; 88 per group)',fs=10.0)

arrow(top,ex); arrow(ex,inc)

xs=[.025,.35,.675]; widths=[.30,.30,.30]

gboxes=[]; bmboxes=[]; fuboxes=[]

for x,w,g in zip(xs,widths,G):

b=box(x,.47,w,.075,f'{SHORT[g]}\nAssigned n = 88\nTime-to-event analysis n = 88',fs=9.3)

gboxes.append(b)

for b in gboxes:

arrow(inc,b,start_x=inc[0]+inc[2]/2,end_x=b[0]+b[2]/2)

for x,w,g,b in zip(xs,widths,G,gboxes):

comp=int(df.loc[df.group.eq(g),'biomarker_complete'].sum()); miss=88-comp

bb=box(x,.315,w,.065,f'3-month biomarkers\nComplete n = {comp}; missing n = {miss}',fs=9.0)

bmboxes.append(bb); arrow(b,bb)

mace=int(df.loc[df.group.eq(g),'MACE_event'].sum())

complete=int(df.loc[df.group.eq(g),'complete_18m_endpoint_ascertainment'].sum())

lost=int(df.loc[df.group.eq(g),'lost_to_followup'].sum())

death=int(df.loc[df.group.eq(g),'noncardiac_death'].sum())

cc=box(x,.12,w,.105,f'18-month follow-up\nFirst MACE: {mace}\nNon-cardiac death: {death}; lost: {lost}\nComplete ascertainment: {complete}',fs=8.55)

fuboxes.append(cc); arrow(bb,cc)

ax.text(.5,.065,'All 264 participants contributed time at risk until first MACE or censoring.',

ha='center',va='center',fontsize=10.0,fontweight='bold')

ax.text(.5,.025,'Quota closure dates in the supplied enrolment file: control 22 Nov 2022; spirulina 31 Jan 2023; diet + spirulina 9 Jun 2023.',

ha='center',va='center',fontsize=7.8)

savefig(fig,'Figure_1_Participant_Flow')

\# Figures 2-4 change plots with jitter, median/IQR boxes

rng=np.random.default_rng(42)

def change_panel(keys,name,folder=FIG,ncols=None):

n=len(keys)

if ncols is None: ncols=3 if n==3 else 2

rows=int(math.ceil(n/ncols))

fig,axes=plt.subplots(rows,ncols,figsize=(7.1,2.75*rows),squeeze=False,

constrained_layout=True)

axes=axes.ravel()

panel_letters='ABCDEFGHIJKLMNOPQRSTUVWXYZ'

for idx,(ax,k) in enumerate(zip(axes,keys)):

b,m,label,unit,_=outs[k]

for j,g in enumerate(G):

vals=(df.loc[df.group.eq(g)&df.biomarker_complete.eq(1),m]-

df.loc[df.group.eq(g)&df.biomarker_complete.eq(1),b]).dropna().values

x=j+rng.normal(0,.045,len(vals))

ax.scatter(x,vals,s=8,alpha=.45)

q1,med,q3=np.quantile(vals,[.25,.5,.75])

ax.plot([j-.16,j+.16],[med,med],linewidth=2.2)

ax.plot([j,j],[q1,q3],linewidth=5,solid_capstyle='butt')

ax.axhline(0,linewidth=.75,linestyle='--')

ax.set_xticks(range(3),[SHORT[g] for g in G],rotation=20,ha='right')

ax.set_ylabel(f'Change from baseline ({unit})')

ax.set_title(f'{panel_letters[idx]}. {label}',loc='left',fontweight='bold',pad=6)

ax.spines[['top','right']].set_visible(False)

for ax in axes[n:]: ax.axis('off')

savefig(fig,name,folder)

change_panel(['ALB','TP','MNA'],'Figure_2_Nutritional_Change',ncols=3)

change_panel(['TC','TG','HDL','LDL'],'Figure_3_Lipid_Change',ncols=2)

change_panel(['Hcy','NO','ET1'],'Figure_4_Homocysteine_Endothelial_Change',ncols=3)

\# Figure 5 KM with risk table and early censor counts

fig=plt.figure(figsize=(7.1,5.3)); gs=fig.add_gridspec(2,1,height_ratios=[3.6,1.4],hspace=.18); ax=fig.add_subplot(gs[0]); rt=fig.add_subplot(gs[1])

for g in G:

cur=km[g]; x=[z[0]/30 for z in cur]; y=[z[1] for z in cur]; ax.step(x,y,where='post',label=SHORT[g])

d=df[(df.group.eq(g))&(df.MACE_event.eq(0))&(df.event_or_censor_day<540)]

for t in d.event_or_censor_day:

ax.plot(t/30,step_at(cur,t),marker='|',markersize=5)

ax.set_xlim(0,18); ax.set_ylim(.65,1.01); ax.set_xlabel('Months from enrolment'); ax.set_ylabel('MACE-free survival probability'); ax.legend(frameon=False,loc='lower left'); ax.grid(axis='y',linewidth=.35,alpha=.4)

times=[0,3,6,9,12,15,18]; rt.axis('off'); rt.set_xlim(-4.1,18.4); rt.set_ylim(-.2,3.8)

rt.text(-4.0,3.35,'Number at risk',fontweight='bold')

for t in times: rt.text(t,3.35,str(t),ha='center',fontweight='bold')

for i,g in enumerate(G):

y=2.55-i*.9; rt.text(-4.0,y,SHORT[g],ha='left');

for t in times: rt.text(t,y,str(int((df.loc[df.group.eq(g),'event_or_censor_day']>=t*30).sum())),ha='center')

rt.text(-4.0,.05,'Vertical ticks indicate censoring before 18 months; administrative censoring at day 540 is not shown.',fontsize=6.8)

savefig(fig,'Figure_5_MACE_Free_Survival')

\# Supplementary Figure S1 love plot

bb=balance_df.sort_values('max_smd_before',ascending=True)

fig,ax=plt.subplots(figsize=(7.1,7.6)); y=np.arange(len(bb)); ax.scatter(bb.max_smd_before,y,label='Unweighted',marker='o'); ax.scatter(bb.max_smd_after,y,label='Generalized-overlap weighted',marker='s'); ax.axvline(.1,linestyle='--',linewidth=1); ax.set_yticks(y,[x.replace('_',' ') for x in bb.variable]); ax.set_xlabel('Maximum absolute standardized mean difference'); ax.legend(frameon=False); fig.tight_layout(); savefig(fig,'Figure_S1_Covariate_Balance',SUPFIG)

\# S2 propensity score overlap

fig,axes=plt.subplots(1,3,figsize=(7.1,2.6),sharey=True)

for k,(ax,g) in enumerate(zip(axes,G)):

for hgroup in G:

ax.hist(ps[df.group.eq(hgroup),k],bins=14,alpha=.45,label=SHORT[hgroup])

ax.set_title(f'Probability of {SHORT[g]}'); ax.set_xlabel('Estimated probability')

axes[0].set_ylabel('Participants'); axes[-1].legend(frameon=False,fontsize=6); fig.tight_layout(); savefig(fig,'Figure_S2_Propensity_Overlap',SUPFIG)

\# S3 competing risk CIF

fig,ax=plt.subplots(figsize=(7.1,4.2))

for g in G:

cur=aj[g]; ax.step([z[0]/30 for z in cur],[z[2] for z in cur],where='post',label=SHORT[g])

ax.set_xlim(0,18); ax.set_ylim(0,.35); ax.set_xlabel('Months from enrolment'); ax.set_ylabel('Cumulative incidence of first MACE'); ax.legend(frameon=False); ax.grid(axis='y',linewidth=.35,alpha=.4); fig.tight_layout(); savefig(fig,'Figure_S3_Competing_Risk_CIF',SUPFIG)

\# S4 weights

fig,ax=plt.subplots(figsize=(7.1,3.6)); data=[ow[df.group.eq(g)] for g in G]; ax.boxplot(data,labels=[SHORT[g] for g in G],showfliers=True); ax.axhline(1,linestyle='--',linewidth=.8); ax.set_ylabel('Normalized generalized-overlap weight'); fig.tight_layout(); savefig(fig,'Figure_S4_Weight_Distribution',SUPFIG)

\# S5 raw baseline vs 3m small multiples

fig,axes=plt.subplots(2,5,figsize=(7.1,5.0)); axes=axes.ravel()

for ax,(k,(b,m,label,unit,_)) in zip(axes,outs.items()):

for g in G:

d=df[df.group.eq(g)&df.biomarker_complete.eq(1)]; ax.scatter(d[b],d[m],s=5,alpha=.35,label=SHORT[g])

lo=min(df[b].min(),df[m].min()); hi=max(df[b].max(),df[m].max()); ax.plot([lo,hi],[lo,hi],linestyle='--',linewidth=.6); ax.set_title(label,fontsize=7,fontweight='bold'); ax.set_xlabel('Baseline',fontsize=6); ax.set_ylabel('3 months',fontsize=6)

axes[-1].legend(frameon=False,fontsize=5); fig.tight_layout(); savefig(fig,'Figure_S5_Individual_Biomarker_Values',SUPFIG)

print(json.dumps({'outdir':str(OUT),'counts':report['counts'],'km':report['km_risk_18m'],'cox':cox,'max_smd_after':report['max_smd_after'],'ph_global_p':global_ph,'files':len(list(OUT.rglob('*')))},indent=2))

\# =============================================================================

\# SECOND REVISION: analyses added in response to Reviewer 1, comments 1 to 3

\# -----------------------------------------------------------------------------

\# These blocks use the revised deidentified dataset, which adds the enrolment

\# window, product batch and serious-event episode columns. They reproduce

\# Supplementary Tables S12, S14 and S15 and Supplementary Figures S6 to S8.

\# =============================================================================

REV = SCRIPT_DIR / 'Supplementary Data File 1 Deidentified Dataset Revised.csv'

if REV.is_file():

rv = pd.read_csv(REV)

for _c in rv.columns:

if _c not in cat_cols | {'enrolment_window_label', 'product_batch'}:

rv[_c] = pd.to_numeric(rv[_c], errors='coerce')

R2 = OUT / 'Second_Revision'

R2.mkdir(parents=True, exist_ok=True)

\# ---------------------------------------------------------------- comment 1

\# Product-batch accountability and batch sensitivity analyses.

batch_alloc = pd.crosstab(rv.product_batch, rv.group).reindex(columns=G, fill_value=0)

sup = rv[rv.group.ne('CG')]

batch_profile = sup.groupby('product_batch').agg(

n=('id', 'size'),

adherence_mean=('spirulina_adherence_pct', 'mean'),

adherence_ge80_n=('spirulina_adherence_ge80', 'sum'),

any_teae_n=('any_nonserious_TEAE', 'sum'),

gi_n=('GI_discomfort', 'sum')).round(3)

def ancova_subset(outcome_key, data, covs=full_cov, extra=None):

"""ANCOVA on an arbitrary subset, mirroring fit_ancova's specification."""

b, m, _, _, _ = outs[outcome_key]

d = data[data.biomarker_complete.eq(1)]

X = pd.DataFrame({'const': 1.0,

'SP': d.group.eq('SP').astype(float),

'DS': d.group.eq('DS').astype(float)}, index=d.index)

for c in [b] + covs:

X[c] = pd.to_numeric(d[c], errors='coerce')

if extra is not None:

for c, v in extra.items():

X[c] = pd.to_numeric(d[v], errors='coerce')

y = d[m].astype(float)

keep = X.notna().all(axis=1) & y.notna()

X, y = X.loc[keep], y.loc[keep]

X = X[[c for c in X.columns if c == 'const' or X[c].std() > 0]]

fit = sm.OLS(y, X).fit(cov_type='HC3')

res = {}

for name, spec in [('SP-CG', {'SP': 1}), ('DS-CG', {'DS': 1})]:

v = np.zeros(X.shape[1])

for k, val in spec.items():

v[X.columns.get_loc(k)] = val

tt = fit.t_test(v)

est = float(np.asarray(tt.effect).squeeze())

se = float(np.asarray(tt.sd).squeeze())

res[name] = {'estimate': est, 'ci_low': est - 1.96 * se,

'ci_high': est + 1.96 * se, 'p': float(tt.pvalue)}

res['n'] = int(len(y))

res['fit'] = fit

res['X'] = X

return res

\# (a) restrict the supplemented groups to the earliest batch

batch_a = rv[rv.group.eq('CG') | rv.product_batch.eq('A')]

batch_a_res = {k: {c: ancova_subset(k, batch_a)[c] for c in ['SP-CG', 'DS-CG']}

for k in outs}

\# (b) add a batch indicator among supplemented participants

sup_c = rv[rv.group.ne('CG') & rv.biomarker_complete.eq(1)].copy()

sup_c['batch_BC'] = sup_c.product_batch.isin(['B', 'C']).astype(float)

batch_effect, batch_p = {}, []

for k in outs:

b, m, _, _, _ = outs[k]

X = pd.DataFrame({'const': 1.0, 'DS': sup_c.group.eq('DS').astype(float),

'batch_BC': sup_c.batch_BC}, index=sup_c.index)

for c in [b] + [v for v in full_cov if v != 'calendar_period']:

X[c] = pd.to_numeric(sup_c[c], errors='coerce')

y = sup_c[m].astype(float)

keep = X.notna().all(axis=1) & y.notna()

X, y = X.loc[keep], y.loc[keep]

X = X[[c for c in X.columns if c == 'const' or X[c].std() > 0]]

fit = sm.OLS(y, X).fit(cov_type='HC3')

v = np.zeros(X.shape[1])

v[X.columns.get_loc('batch_BC')] = 1

tt = fit.t_test(v)

est = float(np.asarray(tt.effect).squeeze())

se = float(np.asarray(tt.sd).squeeze())

p = float(tt.pvalue)

batch_p.append(p)

batch_effect[k] = {'estimate': est, 'ci_low': est - 1.96 * se,

'ci_high': est + 1.96 * se, 'p': p}

batch_fisher_p = float(stats.combine_pvalues(batch_p, method='fisher').pvalue)

batch_q = dict(zip(outs, bh(batch_p)))

\# ---------------------------------------------------------------- comment 2

\# Sensitivity analysis restricted to the concurrent recruitment window.

conc = rv[rv.concurrent_recruitment_cohort.eq(1)]

conc_rows, conc_p = [], []

for k in outs:

full_est = fit_ancova(k, full_cov)

c_est = ancova_subset(k, conc)

conc_p += [c_est['SP-CG']['p'], c_est['DS-CG']['p']]

conc_rows.append({'outcome': k, 'label': outs[k][2], 'unit': outs[k][3],

'full_SP': full_est['SP-CG'], 'full_DS': full_est['DS-CG'],

'conc_SP': c_est['SP-CG'], 'conc_DS': c_est['DS-CG'],

'n_concurrent': c_est['n']})

conc_q = bh(conc_p)

for i, r in enumerate(conc_rows):

r['q_SP'] = float(conc_q[2 * i])

r['q_DS'] = float(conc_q[2 * i + 1])

conc_df = pd.DataFrame([{

'outcome': r['label'], 'unit': r['unit'],

'full_SP': r['full_SP']['estimate'], 'conc_SP': r['conc_SP']['estimate'],

'full_DS': r['full_DS']['estimate'], 'conc_DS': r['conc_DS']['estimate'],

'q_SP': r['q_SP'], 'q_DS': r['q_DS']} for r in conc_rows]).round(4)

_, _, conc_cox = cox_fit(conc)

conc_counts = conc.groupby('group').agg(n=('id', 'size'),

mace=('MACE_first_episode', 'sum'))

excluded = rv[rv.concurrent_recruitment_cohort.eq(0)]

excl_counts = excluded.groupby('group').agg(n=('id', 'size'),

mace=('MACE_first_episode', 'sum'))

\# ---------------------------------------------------------------- comment 3

\# Reconciliation of serious adverse events: participants versus episodes.

def newcombe_rd(a, n1, b, n2, alpha=0.05):

z = stats.norm.ppf(1 - alpha / 2)

def wilson(x, n):

p = x / n

den = 1 + z ** 2 / n

cen = p + z ** 2 / (2 * n)

hw = z * np.sqrt(p * (1 - p) / n + z ** 2 / (4 * n ** 2))

return (cen - hw) / den, (cen + hw) / den

l1, u1 = wilson(a, n1)

l2, u2 = wilson(b, n2)

p1, p2 = a / n1, b / n2

d = p1 - p2

return (100 * d,

100 * (d - np.sqrt((p1 - l1) ** 2 + (u2 - p2) ** 2)),

100 * (d + np.sqrt((u1 - p1) ** 2 + (p2 - l2) ** 2)))

recon = []

for g in G:

d = rv[rv.group.eq(g)]

py = float(d.event_or_censor_day.sum()) / 365.25

episodes = int(d.serious_event_episodes_total.sum())

recon.append({

'group': g,

'participants_first_MACE': int(d.MACE_first_episode.sum()),

'first_MACE_episodes': int(d.MACE_first_episode.sum()),

'recurrent_MACE_episodes': int(d.MACE_recurrent_episodes.sum()),

'total_MACE_episodes': int(d.MACE_episodes_total.sum()),

'participants_nonMACE_SAE': int(d.nonMACE_SAE.sum()),

'nonMACE_SAE_episodes': int(d.nonMACE_SAE_episodes.sum()),

'total_serious_event_episodes': episodes,

'participants_both_categories': int(

((d.MACE_first_episode == 1) & (d.nonMACE_SAE == 1)).sum()),

'unique_participants_any_serious_event': int(

d.any_serious_event_participant.sum()),

'person_years': round(py, 1),

'episodes_per_100_person_years': round(100 * episodes / py, 1)})

recon_df = pd.DataFrame(recon)

ref = recon_df.loc[recon_df.group.eq('CG'),

'unique_participants_any_serious_event'].iloc[0]

for g in ['SP', 'DS']:

a = recon_df.loc[recon_df.group.eq(g),

'unique_participants_any_serious_event'].iloc[0]

rd, lo, hi = newcombe_rd(int(a), 88, int(ref), 88)

recon_df.loc[recon_df.group.eq(g), 'rd_vs_control_pp'] = round(rd, 1)

recon_df.loc[recon_df.group.eq(g), 'rd_ci_low'] = round(lo, 1)

recon_df.loc[recon_df.group.eq(g), 'rd_ci_high'] = round(hi, 1)

\# internal consistency assertion: episodes must equal the sum of components

assert (recon_df.first_MACE_episodes + recon_df.recurrent_MACE_episodes

+ recon_df.nonMACE_SAE_episodes

== recon_df.total_serious_event_episodes).all()

\# ------------------------------------------------------------------ export

batch_alloc.to_csv(R2 / 'S12_batch_allocation.csv')

batch_profile.to_csv(R2 / 'S12_batch_profile.csv')

pd.DataFrame(batch_effect).T.assign(

q=[batch_q[k] for k in batch_effect]).to_csv(R2 / 'S12_batch_effect.csv')

pd.crosstab(rv.enrolment_window_label, rv.group).to_csv(R2 / 'S13_enrolment_timeline.csv')

conc_df.to_csv(R2 / 'S14_concurrent_biomarkers.csv', index=False)

recon_df.to_csv(R2 / 'S15_serious_event_reconciliation.csv', index=False)

json.dump({'batch_fisher_p': batch_fisher_p,

'concurrent_cox': conc_cox,

'concurrent_counts': conc_counts.to_dict(),

'excluded_counts': excl_counts.to_dict(),

'batch_a_restricted': {k: {c: batch_a_res[k][c] for c in batch_a_res[k]}

for k in batch_a_res}},

open(R2 / 'second_revision_summary.json', 'w'), indent=2, default=float)

print(json.dumps({'second_revision_outputs': str(R2),

'batch_fisher_p': round(batch_fisher_p, 4),

'concurrent_HR_SP': round(conc_cox['SP']['hr'], 3),

'concurrent_HR_DS': round(conc_cox['DS']['hr'], 3),

'serious_event_episodes': recon_df.total_serious_event_episodes.tolist(),

'unique_participants': recon_df.unique_participants_any_serious_event.tolist()},

indent=2))
